# The bacterial effector SidN/Lpg1083 promotes cell death by targeting Lamin-B2

**DOI:** 10.1093/jmcb/mjad036

**Published:** 2023-05-30

**Authors:** Jiajia Gao, Wenwen Xu, Feng Tang, Minrui Xu, Qin Zhou, Xingyuan Yang, Nannan Zhang, Jinming Ma, Qi Yang, Xiaofang Chen, Ximing Qin, Honghua Ge

**Affiliations:** Institute of Health Sciences and Technology, Institutes of Material Science and Information Technology, Anhui University, Hefei 230601, China; Institute of Health Sciences and Technology, Institutes of Material Science and Information Technology, Anhui University, Hefei 230601, China; Institute of Health Sciences and Technology, Institutes of Material Science and Information Technology, Anhui University, Hefei 230601, China; Institute of Health Sciences and Technology, Institutes of Material Science and Information Technology, Anhui University, Hefei 230601, China; Institute of Health Sciences and Technology, Institutes of Material Science and Information Technology, Anhui University, Hefei 230601, China; Institute of Health Sciences and Technology, Institutes of Material Science and Information Technology, Anhui University, Hefei 230601, China; Institute of Health Sciences and Technology, Institutes of Material Science and Information Technology, Anhui University, Hefei 230601, China; School of Life Sciences, Anhui University, Hefei 230601, China; Institute of Health Sciences and Technology, Institutes of Material Science and Information Technology, Anhui University, Hefei 230601, China; Institute of Health Sciences and Technology, Institutes of Material Science and Information Technology, Anhui University, Hefei 230601, China; Institute of Health Sciences and Technology, Institutes of Material Science and Information Technology, Anhui University, Hefei 230601, China; Institute of Health Sciences and Technology, Institutes of Material Science and Information Technology, Anhui University, Hefei 230601, China; Institute of Health Sciences and Technology, Institutes of Material Science and Information Technology, Anhui University, Hefei 230601, China; School of Life Sciences, Anhui University, Hefei 230601, China; Information Materials and Intelligent Sensing Laboratory of Anhui Province, Anhui University, Hefei 230601, China

**Keywords:** Lamin-B2, Importin-13, cell death, *Legionella pneumophila*, SidN, Lpg1083, T4SS effector

## Abstract

To facilitate survival, replication, and dissemination, the intracellular pathogen *Legionella pneumophila* relies on its unique type IVB secretion system (T4SS) to deliver over 330 effectors to hijack host cell pathways in a spatiotemporal manner. The effectors and their host targets are largely unexplored due to their low sequence identity to the known proteins and functional redundancy. The T4SS effector SidN (Lpg1083) is secreted into host cells during the late infection period. However, to the best of our knowledge, the molecular characterization of SidN has not been studied. Herein, we identified SidN as a nuclear envelope-localized effector. Its structure adopts a novel fold, and the N-terminal domain is crucial for its specific subcellular localization. Furthermore, we found that SidN is transported by eukaryotic karyopherin Importin-13 into the nucleus, where it attaches to the N-terminal region of Lamin-B2 to interfere with the integrity of the nuclear envelope, causing nuclear membrane disruption and eventually cell death. Our work provides new insights into the structure and function of an *L. pneumophila* effector protein, and suggests a potential strategy utilized by the pathogen to promote host cell death and then escape from the host for secondary infection.

## Introduction

*Legionella pneumophila*, the causative agent of Legionnaires’ disease, is a Gram-negative facultative intracellular pathogen capable of multiplying in a wide spectrum of eukaryotic cells. The infection and subsequent pathology require the defect in organelle trafficking/intracellular multiplication (Dot/Icm) type IVB secretion system (T4SS), whose primitive function is to transfer DNA by bacterial conjugation (Vogel et al., 1998). Through this unique secretion system, over 330 bacterial proteins called *Legionella* effectors are injected into host cells (Mondino et al., 2020). These effectors modulate a variety of host cellular processes, such as endocytic maturation, vesicle trafficking, ubiquitylation, gene expression, dendritic cell formation, lipid metabolism, and apoptotic pathways, to benefit the survival, replication, and egress of *L. pneumophila* (Qiu and Luo, 2017; Schroeder, 2017).

It is obviously important for pathogens to avoid host cell death during intracellular replication at the beginning of infection. However, pathogens are able to induce cell death to their own advantage, particularly to escape from host cells for dissemination (Broz et al., 2012). To date, at least seven *L. pneumophila* T4SS effectors have been found to be involved in the modulation of cell death pathways (Mondino et al., 2020). Previous studies demonstrated that SidF interacts with and neutralizes proapoptotic BNIP3 and Bcl-rambo, while SdhA contributes to the prevention of cell death through a currently unknown mechanism (Laguna et al., 2006; Banga et al., 2007; Nogueira et al., 2009; Ge et al., 2012). In contrast to SdhA and SidF, several effectors (VipD, LegS2, Lem12, Ceg18, and Lpg0716) have been proved to trigger host cell death via caspase-3 activation (Zhu et al., 2013). For example, VipD, a phospholipase, hydrolyzes phosphocholine and phosphatidylethanolamine to destabilize the mitochondrial membrane, leading to the release of cytochrome c, caspase-3 activation, and cell death. However, deficiency in essential apoptotic executioners does not affect *L. pneumophila*-induced cell death, suggesting the possibility that the pathogen activates multiple programmed cell death pathways to facilitate bacterial egress (Speir et al., 2017). This indicates that undefined effector(s) may induce other distinct cell death pathways in late stages of *L. pneumophila* infection.

SidN (Lpg1083) is one of the *L. pneumophila* T4SS effectors and does not exhibit sequence homology with any functionally annotated proteins. A previous study mentioned that SidN was preferably expressed in the transmissive phase (Bruggemann et al., 2006), which implies a role in assisting the dissemination of *L. pneumophila*. Here, we incorporated X-ray crystallography, cellular imaging, and biochemistry to investigate whether and how SidN affects mammalian cells. Our results show that SidN is a nucleus-localized effector, and its structure adopts a novel fold. When ectopically expressed in mammalian cells, SidN is transported by Importin-13 into the nucleus, where it attaches to Lamin-B2 and interferes with structural integrity of the nuclear envelope architecture, leading to nuclear membrane disruption and eventually cell death.

## Results

### SidN exhibits toxicity to mammalian cells

**Figure 1:**
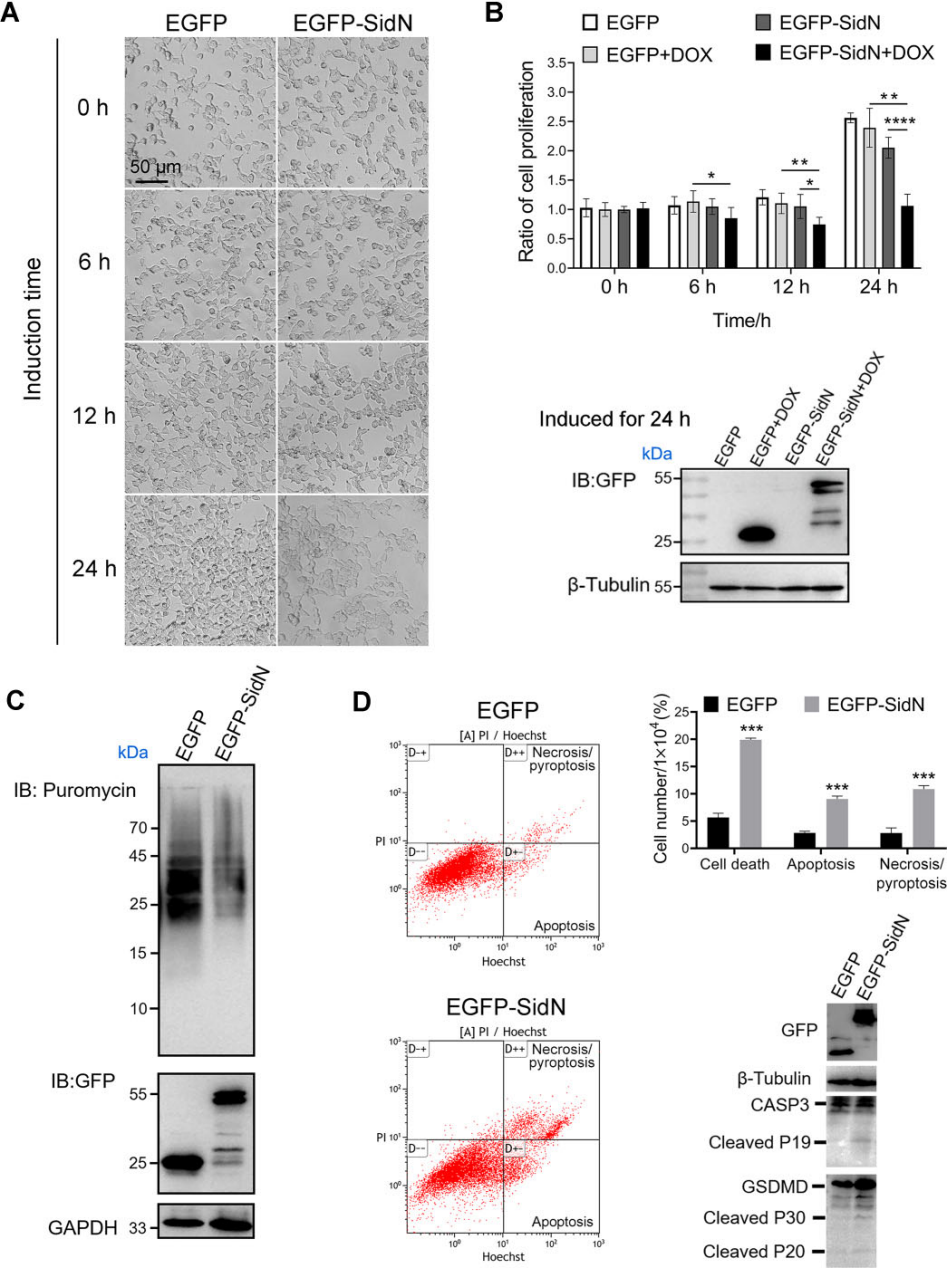
SidN exhibits toxicity to mammalian cells. (**A** and **B**) SidN inhibits HEK293T cell growth and proliferation. HEK293T cells were induced by doxycycline for the indicated periods to express EGFP or EGFP-SidN. (**A**) Cell growth was monitored under a bright-field microscope. (**B**) The relative proliferation ratio was determined by the MTT assay. The expression of EGFP and EGFP-SidN was detected by immunoblotting with an anti-GFP antibody. β-Tubulin was used as a loading control. DOX, doxycycline. (**C**) SidN inhibits protein translation in HEK293T cells. Puromycin was added into HEK293T cell culture. After 10 min, puromycin-labeled proteins in the cells were detected by immunoblotting with an anti-puromycin antibody. GAPDH was used as a loading control. (**D**) SidN induces cell death in HEK293T cells. Flow cytometric analysis of cell death was performed. The ratios of apoptosis, necrosis/pyroptosis, and total cell death were calculated. The supernatants of cell lysates were used to detect the cleavage of caspase-3 and GSDMD by western blotting. Images or blots shown are representative of three independent experiments with similar results. Data are shown as mean ± SD. *P < 0.05, **P < 0.01, ***P < 0.001, and ****P < 0.0001 were determined by *t*-test.

SidN, a hypothetical protein with unknown functions, is translocated into host cells in a Dot/Icm-dependent manner. To assess the effect of SidN expression on mammalian cell proliferation, we used a doxycycline-inducible expression vector to express EGFP or EGFP-SidN in HEK293T cells. We found that the expression of EGFP-SidN inhibited the growth of HEK293T cells compared to the expression of EGFP (Figure 1A). Methylthiazolyldiphenyl-tetrazolium bromide (MTT) colorimetric assays showed that the number of cells transfected with EGFP-SidN decreased by 25% after 12 h of doxycycline induction in comparison to that without induction, whereas the number of cells transfected with EGFP did not significantly change after induction (Figure 1B). More strikingly, the number of cells transfected with EGFP-SidN decreased by 50% after 24 h of induction in comparison to that without induction (Figure 1B).

In order to better understand the cytotoxic effect of SidN on eukaryotic cells, we performed the *in vivo* puromycylation assay to monitor protein translation. The recombinant EGFP or EGFP-SidN plasmids were transiently transfected into HEK293T cells. After 24 h, the cells were incubated with puromycin for 10 min, and puromycin-incorporated proteins in the cells were detected by immunoblotting with a mouse monoclonal anti-puromycin antibody. The results showed that the amount of new polypeptide chains in the cells was greatly reduced after overexpression of SidN (Figure 1C). Alongside, we checked the effect of SidN on cell death. Compared to transient expression of EGFP, expression of EGFP-SidN in HEK293T cells resulted in a significant increase in cell death within 24 h, accompanied by the cleavage of caspase-3 and GSDMD (Figure 1D). Similar results were observed in different mammalian cell lines transfected with EGFP or EGFP-SidN (Supplementary Figure S1). Taken together, the ectopic expression of EGFP-SidN strongly inhibits host protein synthesis and reduces cell viability compared to the expression of EGFP.

### Crystal structure of SidN

**Figure 2:**
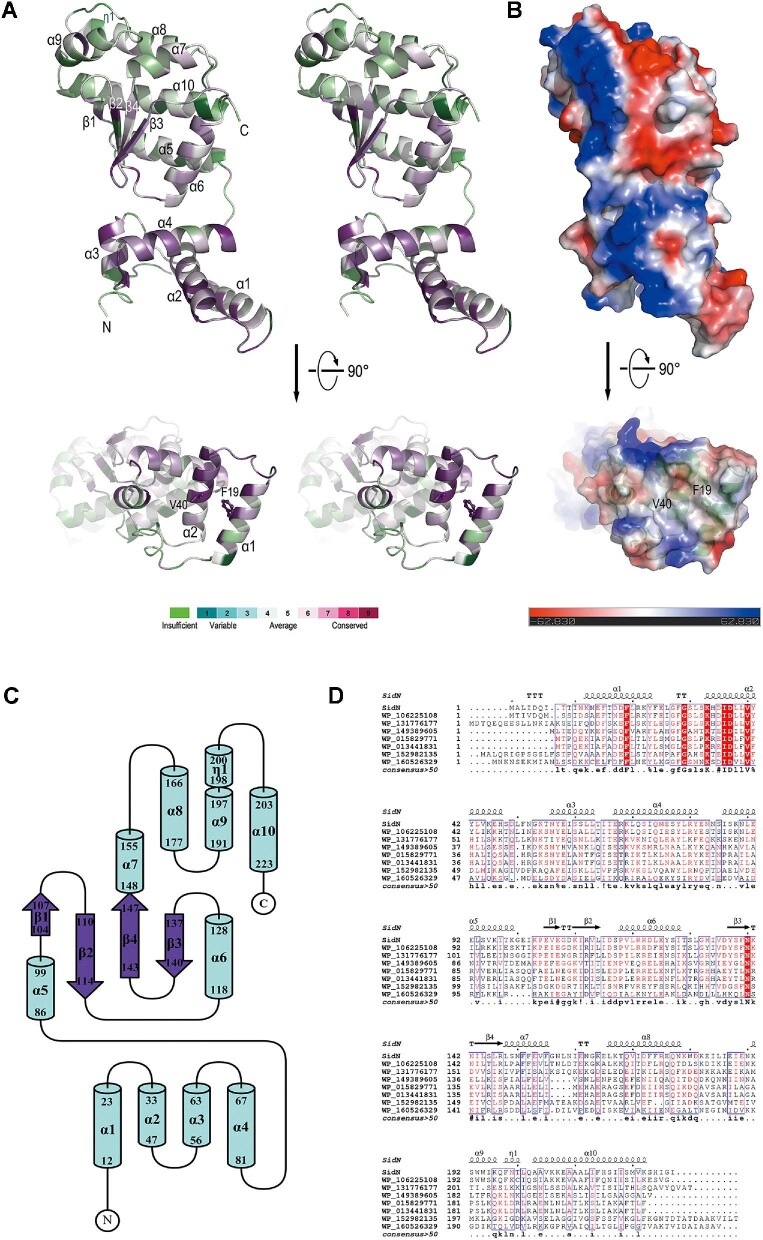
Crystal structure of SidN. (**A**) Stereo view of the ribbon representation of the SidN structure. The conservation pattern was obtained using the Consurf server (Ashkenazy et al., 2016). The correspondence between conservation and color is labeled. The view differs by a 90° rotation about the horizontal axis. The relevant residues are labeled and shown in stick form. (**B**) Electrostatic potential surface plots of SidN. Surface electrostatic potential map of SidN generated by PyMol (v0.99), with positive and negative regions in blue and red, respectively. The structure is represented on the surface at the same orientation as in **A**. (**C**) Topological diagram of SidN. Helices and strands are shown as bars and arrows, respectively. Topology diagrams were generated with Topdraw (Bond, 2003). (**D**) Sequence alignment of SidN and its homologs. The sequence of SidN from *L. pneumophila* (Lpg1083, PDB code 7YJI; this study) was aligned with the sequences of WP_106225108 from *L. pneumophila*, WP_131776177 from *Legionella impletisoli*, WP_149389605 from *Nitrincola tapanii*, WP_015829771 from *Methylovorus glucosotrophus*, WP_013441831 from *Methylovorus sp. MP688*, WP_152982135 from *Prosthecomicrobium hirschii*, and WP_160526329 from *Sphaerochaeta halotolerans.* The alignment was performed using MultAlin (Corpet, 1988) and ESPript (Robert and Gouet, 2014). α-helices, β-strands, and 3_10_-helices are denoted by Greek characters α, β, and η, respectively. Strictly conserved residues are highlighted with red boxes.

In order to obtain clues about its function, we determined the crystal structure of SidN at 2.1 Å resolution by using the single-wavelength anomalous diffraction method. The SidN molecule forms a nearly cylindrical shape with a length of ∼60 Å and a diameter of ∼35 Å (Figure 2A and B). The architecture folds into two domains. The smaller N-terminal paw-like domain (SidN_1–82_) is exclusively α-helical and formed by four helices (α1–α4). The larger C-terminal domain (SidN_83–227_) is composed of six α-helices (α5–α10), one 3_10_ helix (η1), and a four-stranded anti-parallel β-sheet spatially arranged in the order β1/β2/β4/β3 from left to right when facing the sheet. Three anti-parallel helices (α5, α10, and α8) are packed against the β-sheet and the rest helices (α6, α7, α9, and η1). The two domains are connected by a very short loop. The C-terminal domain contacts the paw-like domain via the N-terminus of α6, as well as the loop between β2 and α6 (Figure 2A and C).

The DALI program (Holm and Rosenstrom, 2010) was used to search three-dimensional structural homology to SidN, SidN_1–82_, and SidN_83–227_. The best match (*Z*-score: 5.2) for both SidN and SidN_1–82_ was the structure of the transcription factor PF0095 from *Pyrococcus furiosus* (PDB code 2QLZ). Superposition of the structures showed that three α-helices in the N-terminal domain of SidN adopt a similar fold in spatial arrangement to the relevant region on 2QLZ (Supplementary Figure S2A). The best match (*Z*-score: 4.8) for SidN_83–227_ was the structure of a cobalt energy-coupling factor transporter from *Rhodobacter capsulatus* (PDB code 5X41). Superposition of the structures showed only two α-helices from SidN_83–227_ with similar folding to the relevant region on 5X41 (Supplementary Figure S2B). However, these analogous regions have notable differences in physicochemical properties, thus not providing convincing clue for the function of SidN. Not surprisingly, we did not find arrangements of residues similar to the known active sites. However, the surface of SidN has a hydrophobic concave region lined with conserved residues on the end of the paw-like domain (Figure 2A, B, and D), suggesting that this region may serve as a binding hub.

### SidN co-localizes with the nuclear envelope in mammalian cells

Generally, bacterial effectors exert their functions at specific locations within host cells. To provide insight into the function of SidN in HEK293T cells, we investigated the subcellular localization of SidN. Full-length SidN was N-terminally fused with EGFP and overexpressed in HEK293T cells. Cells overexpressing EGFP served as a control.

**Figure 3:**
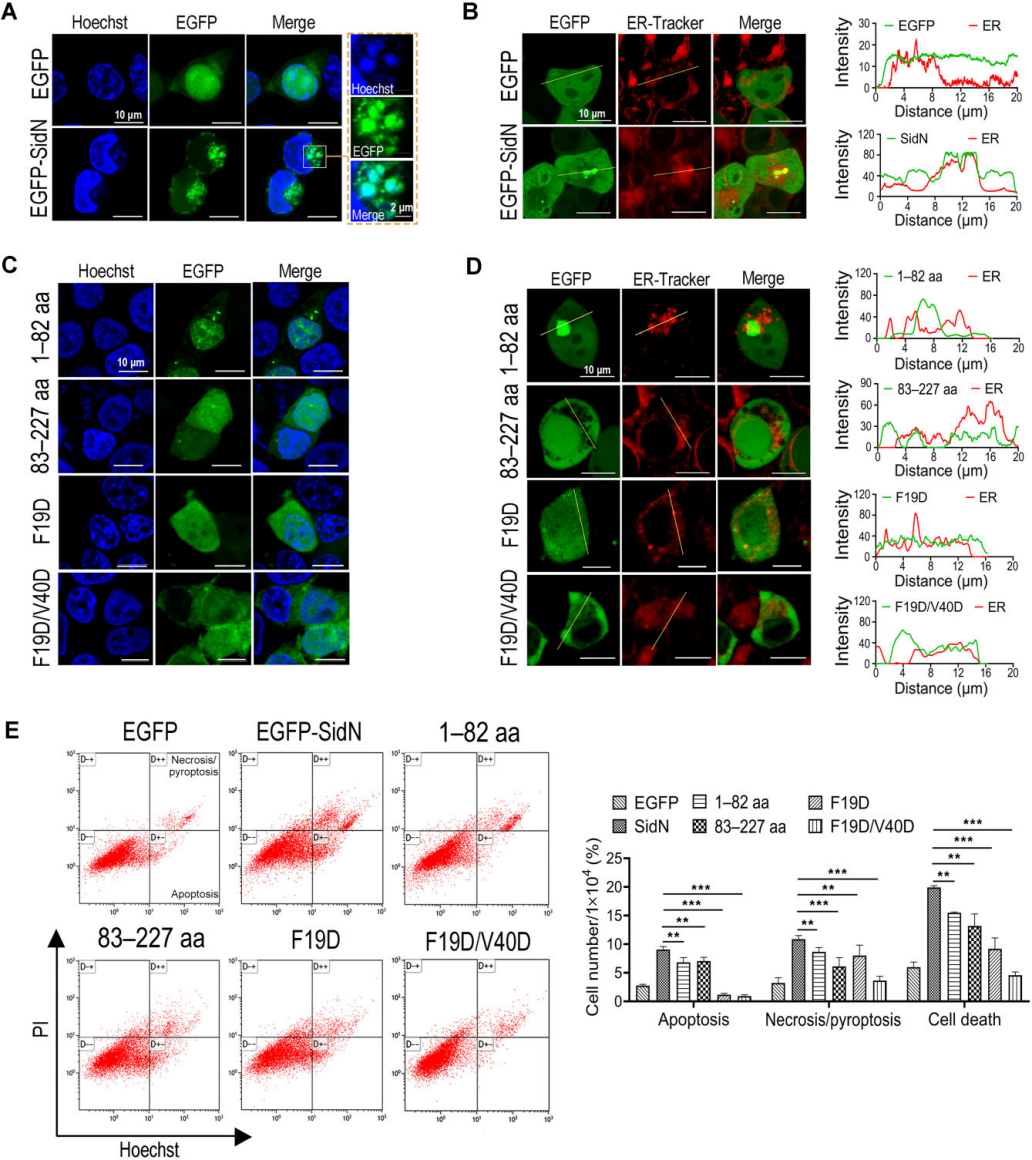
Subcellular localization and cell toxicity of SidN and its mutants. (**A**–**D**) HEK293T cells were induced for 24 h to express EGFP, EGFP-SidN (**A** and **B**), or EGFP-tagged SidN mutants (**C** and **D**). The intracellular localization of SidN and SidN mutants (**A** and **C**) and the co-localization of SidN mutants with the ER (**B** and **D**) were observed by confocal fluorescence microscopy. EGFP indicates the position of EGFP fusion proteins, cell nuclei were visualized with Hoechst stain (blue), and the ER were marked by ER-Tracker (red). Intensity trace analysis was plotted using ImageJ software, and curve coincidence illustrates the co-localization of two proteins in theory. (**E**) Cell death was examined by flow cytometry, and the ratios of apoptosis, necrosis/pyroptosis, and total cell death were calculated. Data are shown as mean ± SD from three independent experiments. **P < 0.01 and ***P < 0.001 were determined by *t*-test.

While EGFP was evenly distributed throughout the cell, EGFP-SidN exhibited an enriched and noticeably concentrated punctate distribution at or near the endoplasmic reticulum (ER) and the nuclear envelope (Figure 3A). The localization of EGFP-SidN and HA-SidN was also investigated in HeLa, A549, PC9, RAW 264.7, PC9, NIH3T3, and HEK293T cells, respectively, and similar results were observed (Supplementary Figure S3A and B). To further identify the relevant subcellular compartment, we stained HEK293T cells expressing EGFP-SidN with ER-Tracker, Emerin, Nup133, and Lamin-A/C, respectively, and observed co-localization at the ER apparatus and probably the nuclear envelope (Figure 3B; Supplementary Figure S3C).

To determine which region is responsible for the localization and lethal effect, two individual domains of SidN were fused with EGFP and expressed in HEK293T cells. The localization of the paw-like domain (SidN_1–82_) was similar to that of full-length SidN, while the C-terminal domain (SidN_83–227_) was evenly distributed throughout the cell without specific localization (Figure 3C and D). This indicates that the characteristic distribution of SidN depends on its paw-like domain.

Additionally, we used site-directed mutagenesis to test whether the hydrophobic region in the paw-like domain is involved in SidN localization. A single-amino acid change of phenylalanine (Phe19) or mutation of both phenylalanine (Phe19) and valine (Val40) in the center of the region to charged polar residues significantly decreased the punctate distribution of SidN at the ER and nuclear envelope (Figure 3C and D), indicating that this conserved hydrophobic patch in the paw-like domain of SidN is essential for subcellular localization. Accordingly, of eukaryotic cell toxicity assays, compared with the expression of EGFP-SidN, the expression of the paw-like domain, the C-terminal domain, SidN^F19D/V40D^, or SidN^F19D^ significantly reduced the toxicity to the cells (Figure 3E).

### Lamin-B2 is a eukaryotic target of SidN

To identify host proteins interacting with SidN and thus unravel the molecular mechanism by which SidN modulates host cell signaling, HEK293T cells overexpressing EGFP-SidN fusion protein were subjected to immunoprecipitation (IP) experiments coupled with protein identification by liquid chromatography–tandem mass spectrometry (LC–MS/MS). Cells overexpressing EGFP were used as a control.

**Figure 4:**
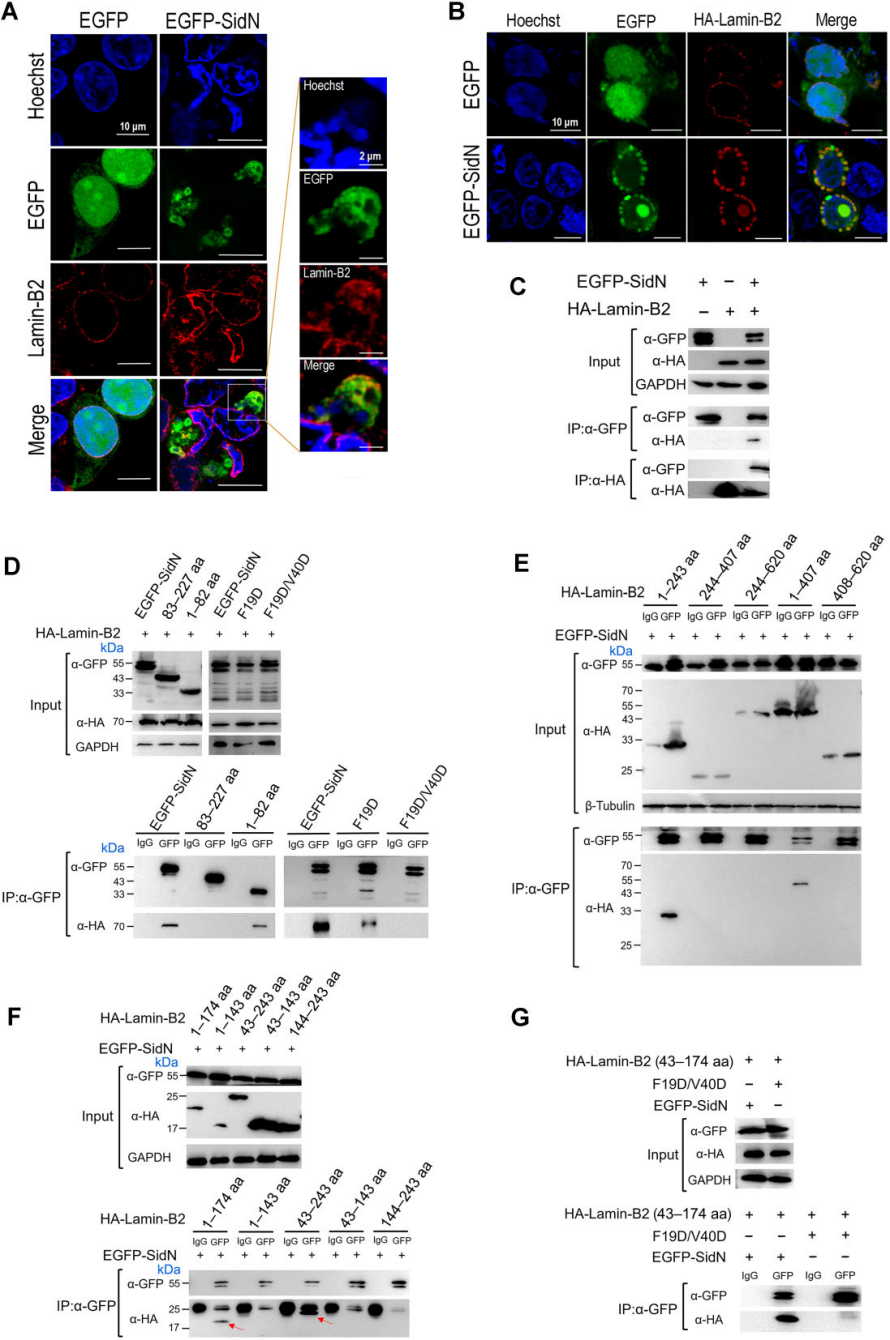
Lamin-B2 is a eukaryotic target of SidN. (**A**) Co-localization of ectopically expressed SidN and *in situ* Lamin-B2. Green fluorescence indicates the position of EGFP or EGFP-SidN, cell nuclei were visualized with Hoechst stain (blue), and Cy3-conjugated anti-rabbit IgG and rabbit anti-Lamin-B2 antibodies were used to display *in situ* Lamin-B2 (red). (**B**) Co-localization of ectopically expressed SidN and Lamin-B2. Green fluorescence indicates the position of EGFP or EGFP-SidN, cell nuclei were visualized with Hoechst stain (blue), and Cy3-conjugated anti-rabbit IgG and rabbit anti-HA antibodies were used to show the position of HA-Lamin-B2. (**C**) Reciprocal co-IP of SidN and Lamin-B2. (**D**) Co-IP of SidN mutants and Lamin-B2. (**E**–**G**) Co-IP of Lamin-B2 deletion mutants and SidN. The fragment 43–174 of Lamin-B2 is essential for SidN–Lamin-B2 binding, and the F19D/V40D mutant of SidN shows a greatly reduced binding ability. Blots shown are representative of three independent experiments with similar results.

LC–MS/MS identified a number of proteins that appeared to associate with SidN in the immunoprecipitates. Based on the nuclear envelope localization of SidN, we hypothesized that Lamin-B2, a component of the nuclear lamina, is a eukaryotic nuclear-associated target of SidN. To test this possibility, we performed co-localization experiments in cells overexpressing SidN alone and cells co-expressing SidN and Lamin-B2. Confocal fluorescence microscopy revealed co-localization of SidN and Lamin-B2 in a morphologically altered nuclear envelope (Figure 4A). Interestingly, co-expression of SidN and Lamin-B2 generated strong egg-like aggregations along the nuclear envelope, which were absent in control cells (Figure 4B).

To further confirm the interactions between Lamin-B2 and SidN, EGFP-SidN and HA-Lamin-B2 were transfected into HEK293T cells, and reciprocal co-IP experiments were performed. HA-Lamin-B2 was detected in the anti-GFP IP of cells co-transfected with EGFP-SidN, and EGFP-SidN was detected in the anti-HA IP of cells co-transfected with HA-Lamin-B2 (Figure 4C). Similar results were obtained from the reciprocal co-IP of Myc-SidN and HA-Lamin-B2 that Myc-SidN was able to immunoprecipitate HA-Lamin-B2 (Supplementary Figure S4). Furthermore, in the anti-GFP IP of cells co-transfected with HA-Lamin-B2 and EGFP-tagged SidN mutants, SidN_1–82_ was detected, while SidN_83–227_ without the nuclear localization ability was not detected, and the binding of both SidN^F19D/V40D^ and SidN^F19D^ to Lamin-B2 was greatly reduced (Figure 4D).

To map the regions in Lamin-B2 involved in SidN binding, we constructed a series of Lamin-B2 deletion mutants and examined their interactions with SidN. Confocal fluorescence microscopy showed that the punctate co-localization was observed in cells co-expressing SidN together with the individual fragment of Laimin-B2_1–174_, Laimin-B2_1–243_, Laimin-B2_1–407_, or Laimin-B2_43–243_, but not with Laimin-B2_43–143_, Laimin-B2_1–143_, Laimin-B2_144–243_, Laimin-B2_244–407_, Laimin-B2_244–620_, or Laimin-B2_408–620_ (Supplementary Figure S5). Accordantly, IP experiments showed that interactions can only be observed for SidN and Laimin-B2 deletion mutants containing the fragment 43–174 (Figure 4E and F). In addition, destruction of the hydrophobic concave region in SidN (SidN^F19D/V40D^) completely blocked the binding of SidN to the fragment 43–174 of Lamin-B2 (Figure 4G).

Taken together, these results strongly suggest the association of SidN with Lamin-B2, which is an important nuclear intermediate filament protein. The fragment 43–174 of Lamin-B2 and the N-terminal domain of SidN are required for the interaction of these two proteins, which likely determines the subcellular localization of SidN.

### SidN is a nuclear effector transported via Importin-13

**Figure 5:**
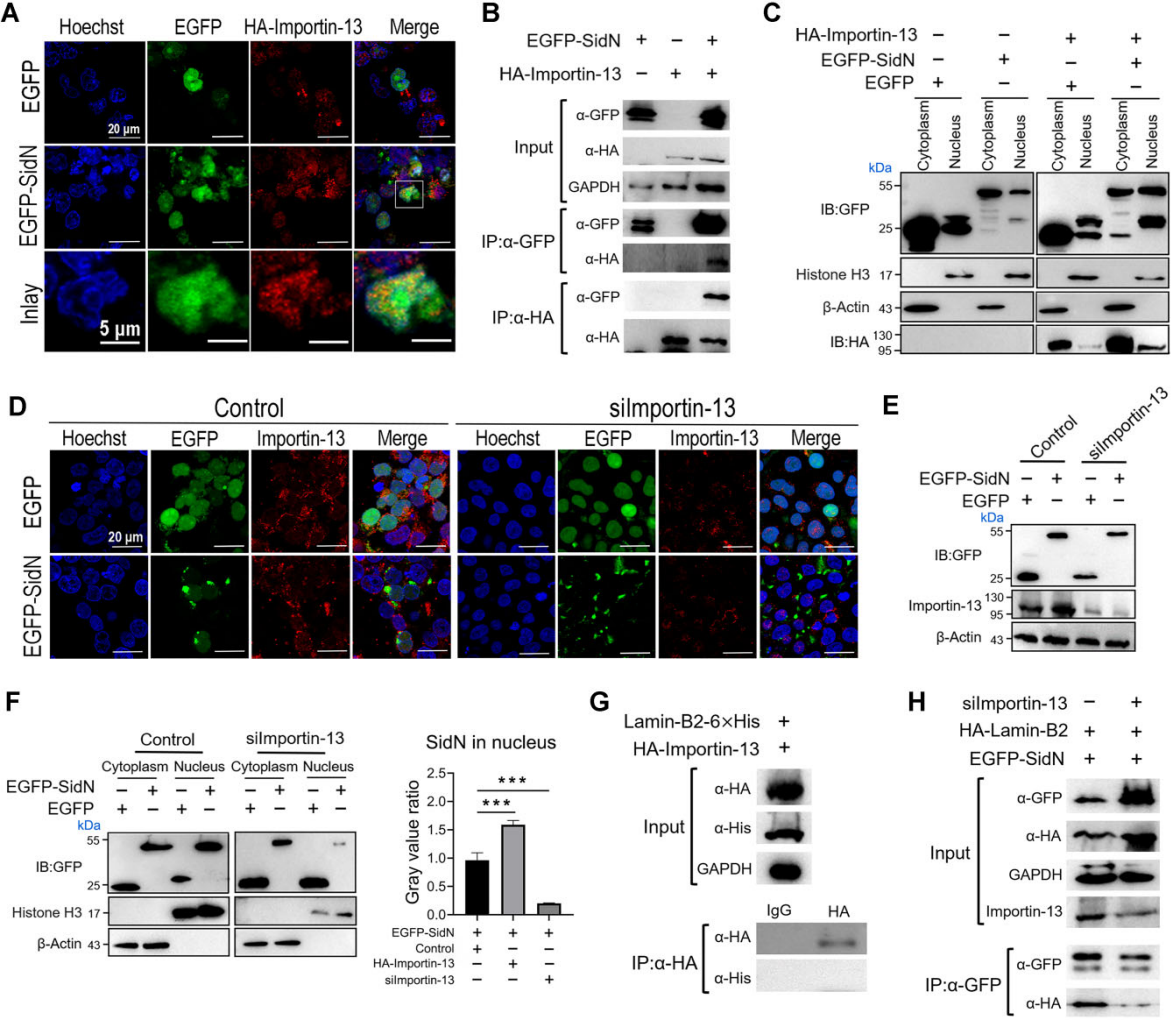
The nuclear import of SidN is mediated by Importin-13. (**A**–**F**) Overexpression of Importin-13 increases the amount of SidN in the nucleus (**A**–**C**), while knockdown of Importin-13 decreases the amount of SidN entering the nucleus (**D**–**F**). (**A**) Representative co-localization images of HEK293T cells co-expressing EGFP or EGFP-SidN and HA-Importin-13. Green fluorescence indicates the position of EGFP or EGFP-SidN, cell nuclei were visualized with Hoechst stain (blue), and Cy3-conjugated anti-rabbit IgG and rabbit polyclonal anti-HA antibodies were used to display HA-Importin-13 (red). (**B**) Reciprocal co-IP of SidN and Importin-13. (**C**) SidN and Importin-13 expression levels in nuclear and cytoplasmic fractions were detected by immunoblotting at 24 h after transfection. (**D**) Representative co-localization images of HEK293T cells co-transfected with control siRNA or siImportin-13 and EGFP or EGFP-SidN plasmids. (**E** and **F**) SidN expression levels in the whole-cell lysates (**E**) and nuclear and cytoplasmic fractions (**F**) were detected by immunoblotting with an anti-GFP antibody. β-Actin and histone H3 served as internal references for cytoplasmic and nuclear proteins, respectively. The graph shows relative SidN expression levels in the nucleus by quantitative analysis of **C** and **F**. Data are shown as mean ± SD. ***P < 0.001 was determined by *t*-test. (**G**) Co-IP assays showing no interaction between Lamin-B2 and Importin-13. (**H**) Knockdown of Importin-13 reduces the interaction between SidN and Lamin-B2. HEK293T cells were co-transfected with EGFP-SidN, HA-Lamin-B2, and control siRNA or siImportin-13. After 24 h, cell lysates were immunoprecipitated with IgG and anti-GFP antibodies and then analyzed by immunoblotting with anti-GFP and anti-HA antibodies. Blots shown are representative of three independent experiments with similar results.

Next, we determined the possible interaction between SidN and eukaryotic karyopherins. Confocal fluorescence microscopy revealed co-localization of SidN with Importin-13, which is a eukaryotic bidirectional nuclear transporter (Mingot et al., 2001; Gajewska et al., 2021), in cells co-expressing EGFP-SidN and HA-Importin-13 (Figure 5A). Reciprocal co-IP analysis revealed that EGFP-SidN co-immunoprecipitated with HA-Importin-13 and HA-Importin-13 co-immunoprecipitated with EGFP-SidN (Figure 5B). Similar results were obtained from the reciprocal co-IP of Myc-SidN and HA-Importin-13 (Supplementary Figure S4). Analysis of the nuclear and cytoplasmic fractions showed that overexpression of Importin-13 significantly increased the amount of SidN in the nucleus (Figure 5C). To confirm that SidN is transported to the nucleus by Importin-13, we knocked down Importin-13 expression in HEK293T cells using siRNA. Confocal fluorescence microscopy and nuclear/cytoplasmic fraction analysis demonstrated that the amount of EGFP-SidN entering the nucleus was greatly decreased in the Importin-13-knockdown cells (Figure 5D–F). These observations suggest that SidN is most likely transported by Importin-13 into the host nucleus and subsequently interacts with Lamin-B2, which is mainly present in the inner nuclear membrane. Additionally, IP experiments showed that there was no interaction between Lamin-B2 and Importin-13 (Figure 5G) and the binding of SidN to Lamin-B2 was significantly reduced in the Importin-13-knockdown cells (Figure 5H), indicating that SidN is transported to the nucleus in a Lamin-B2-independent manner.

## Discussion

In this study, we investigated the structure and function of the Dot/Icm T4SS effector SidN of *L. pneumophila*. It has been shown that SidN is preferably translocated into the host in the transmissive phase during *L. pneumophila* infection (Bruggemann et al., 2006). Here, we showed that SidN exhibits toxicity to eukaryotic cells, and ectopically expressed SidN localizes at the nuclear envelope of mammalian cells and alters nuclear morphology (Figures 1 and 3; Supplementary Figure S3 and Video S1). We also solved the structure of SidN to a resolution of 2.1 Å. The SidN structure consists of two sequentially arranged domains. Structural analysis showed that the N-terminal paw-like domain contains a concave surface at the end of the nearly tubular structure of SidN. Our study showed that the punctate subcellular localization is determined by the N-terminal domain of SidN, in particular by the hydrophobic concave region (Figure 2). Destruction of this region disrupted the characteristic localization of SidN and led to a broad distribution within the cell. However, SidN-mediated lethal effect on mammalian cells requires both the N-terminal paw-like and C-terminal domains (Figure 3E).

**Figure 6:**
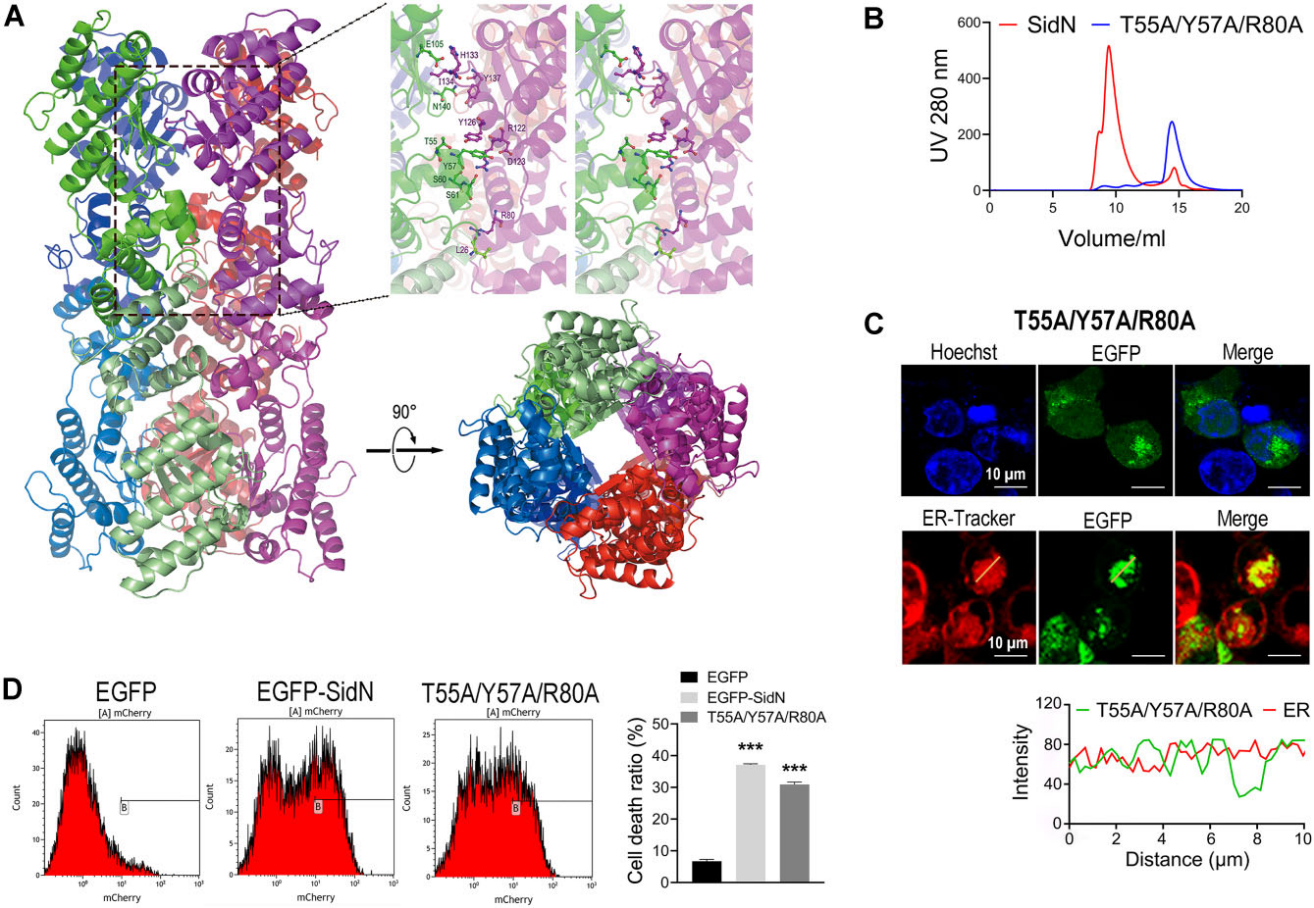
SidN probably exerts its physiological function as a dimer. (**A**) SidN forms an octamer with crystallographic symmetry. The left panel shows the ribbon representation of the octamer with each subunit colored differently. The top right panel shows a close-up view of the interactions between a monomer and its neighboring dimer. The relevant residues are labeled and shown in stick form. In the bottom right panel, the SidN octamer is shown in a different orientation corresponding to 90° rotation along the horizontal axis. (**B**) Oligomeric state determination of SidN and SidN^T55A/Y57A/R80A^. Size-exclusion chromatography characterization showing that SidN^T55A/Y57A/R80A^ eluted as a single peak with an estimated molecular weight of 50 kDa, while SidN eluted as two peaks with estimated molecular weights of 600 kDa and 50 kDa, respectively. (**C**) Subcellular localization of SidN^T55A/Y57A/R80A^. Green fluorescence indicates the position of EGFP-SidN^T55A/Y57A/R80A^, cell nuclei were visualized with Hoechst stain (blue), and the ER were marked by ER-Tracker (red). Intensity trace analysis of SidN^T55A/Y57A/R80A^ and the ER was plotted using ImageJ software. (**D**) Representative flow cytometry results of the apoptosis assay on HEK293T cells transfected with SidN or SidN^T55A/Y57A/R80A^. The ratio death was calculated from three independent experiments. Data are shown as mean ± SD. ***P < 0.001 was determined by *t*-test.

The crystallographic asymmetric unit of the SidN crystal contains one monomer. However, this monomer appears to form a dimer and an octamer with crystallographic symmetry (Figure 6A; Supplementary Figure S3). Analysis using the Protein Interfaces, Structures, and Assemblies (PISA) program (Krissinel and Henrick, 2007) suggests that the SidN dimer and octamer are stable in solution, which is consistent with the dimeric and multimeric states of SidN in size-exclusion chromatography (Figure 6B). Using site-directed mutagenesis, we obtained a stable dimer SidN^T55A/Y57A/R80A^ (Figure 6B), which has a similar phenotype to wild-type SidN in terms of cellular localization and toxicity (Figure 6C and D). This result suggests that octamerization is not essential for SidN activity. According to the dimer structure analysis, the hydrophobic concave region in the N-terminal domain plays a critical role in dimerization (Supplementary Figure S6). Destruction of this region may lead to the inability of SidN to dimerize and consequently lose its intracellular function (Figure 3). Therefore, SidN probably exerts its physiological function as a dimer.

The identification of eukaryotic binding partners may help to elucidate potential functions of SidN in *L. pneumophila* infection. IP experiments coupled with protein identification by LC–MS/MS resulted in a list of potential host target proteins, among which, Lamin-B2, a component of the nuclear lamina, was the most interesting one due to its subcellular co-localization with ectopically expressed SidN. The nuclear lamina is a filamentous meshwork closely associated with the inner nuclear membrane (Dechat et al., 2010). As an intermediate filament-type protein, Lamin-B2 has been found to play important roles in maintaining the integrity of the nuclear skeleton, cell proliferation and aging, gene expression, and DNA damage repair by affecting chromosome distribution, chromatin remodeling, and nuclear membrane rupture and reorganization during mitosis (Dechat et al., 2008; Shimi et al., 2008).

Co-IP coupled with co-localization experiments identified Lamin-B2 as a eukaryotic target of SidN (Figure 4). We observed that SidN was significantly enriched at the nuclear envelope of SidN-transfected cells and interfered with Lamin-B2, thereby altering nuclear morphology (Supplementary Figure S7). Both SidN^F19D/V40D^ and SidN^F19D^ showed the greatly reduced binding to Lamin-B2 (Figure 4D) and a loss of the characteristic punctate subcellular localization (Figure 3C and D), and consequently, a significantly diminished ability to promote cell death (Figure 3E). These observations suggest that the binding of SidN to Lamin-B2 is essential for its toxic effect on cells. Further reciprocal co-IP and co-localization assays suggested that the fragment 43–174 of Lamin-B2 is essential for SidN binding. This segment is conserved in Lamin-A. It is known that lamin proteins exist in the dimeric form prior to filament formation (Kapinos et al., 2010). Recently, two interactions essential for lamin assembly were discovered by crystal structure and biochemical studies (Ahn et al., 2019), which reveals that the analogous region in Lamin-A is involved in lamin dimerization and filament formation. Thus, the fragment 43–174 of Lamin-B2 likely plays a similar role. Moreover, strong egg-like Lamin-B2 aggregates were observed at the periphery of the nuclear envelope in cells co-expressing SidN and Lamin-B2. This is similar to that found in Lamin-A^L59R^-overexpressing cells, in which the L59R mutation destabilizes the coiled-coil interaction that is important for the lamin assembly process (Ahn et al., 2019). Hence, the binding of SidN to the fragment 43–174 of Lamin-B2 probably blocks essential interactions for lamin assembly, leading to a reduction of Lamin-B2 in the nuclear lamina, thereby altering nuclear envelope morphology. This is consistent with a previous study in which Lamin-B2 knockdown severely disrupted robust nuclear structure (Sen Gupta and Sengupta, 2017). Another possibility is that SidN binds to Lamin-B2 and then disrupts other component(s) of the nuclear envelope through a yet unclear mechanism. The nuclear envelope is an important target of the apoptotic machinery (Lindenboim et al., 2020). Thus, alteration in the nuclear envelope structure triggers cell death processes that are responsible for increased cell mortality. Interestingly, SidN co-localizes with the ER before entering the nucleus (Figure 3A and B; Supplementary Video S1), and SidN was shown to inhibit protein synthesis, indicating that SidN may have other targets on the ER. The disruption of the nuclear envelope induced by the SidN–Lamin-B2 interaction may also affect the function of the ER, since it is closely related to the nuclear envelope in structure.

Although bioinformatic analysis of SidN did not identify any canonical nuclear localization signal motifs, co-IP and co-localization experiments showed that SidN may associate with the nuclear transport protein Importin-13. The inhibitory effect of Importin-13 knockdown on the nuclear localization of SidN suggests that Importin-13 may serve as the transporter for delivering SidN into the host nucleus.

**Figure 7:**
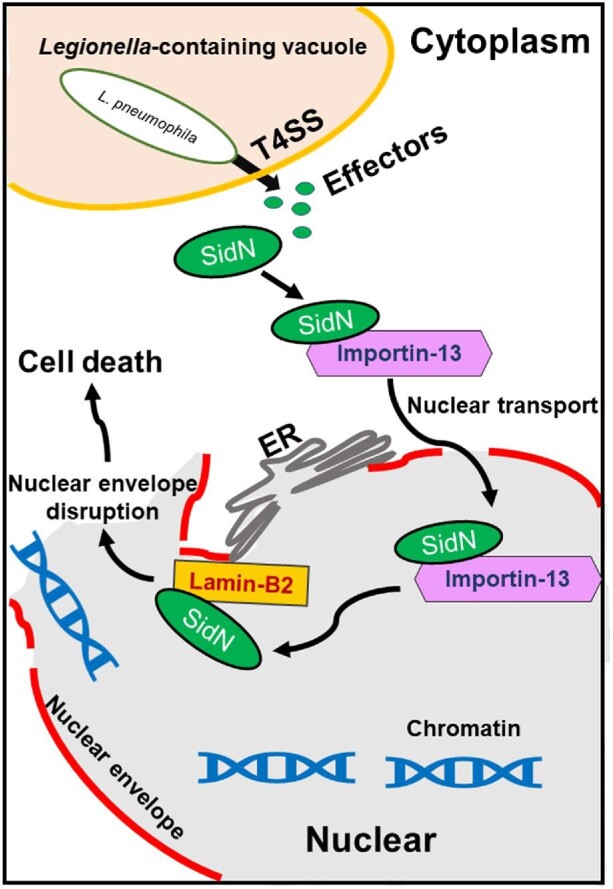
A potential working model of SidN.

Taken together, of over 330 *L. pneumophila* T4SS effectors delivered into host cells, SidN is currently the only effector that exploits a eukaryotic nuclear transporter to target and interfere with the nuclear envelope of mammalian cells. Based on our findings, we proposed a potential working model for the association of SidN with host cell death (Figure 7). In the late stage of *L. pneumophila* infection, SidN is delivered into the host cell and transported into the nucleus by Importin-13. SidN then attaches to Lamin-B2, interferes with the lamina complex, and disrupts the nuclear envelope structure. Subsequent release of host DNA promotes host cell death for bacterial dissemination and secondary infection.

## Materials and methods

### Protein expression and purification

For recombinant expression in *Escherichia coli*, native SidN and the SidN mutants were cloned into the P28 vector (derived from pET-28a by deleting the sequence AGCAGCGGCCTGGTGCCGCGCGGCAGC between the *Nco*I and *Nde*I restriction sites). Recombinant proteins were expressed in *E. coli* strain Rosetta with 0.2 mM isopropyl-D-1-thiogalactopyranoside for 20 h induction at 16°C in Luria–Bertani medium. Selenomethionine-substituted SidN (SeMet-SidN) was overexpressed in the same competent cells as native SidN but using M9 medium based on a methionine-biosynthesis inhibition method (supplemented with SeMet and six amino acids, including threonine, lysine, phenylalanine, valine, leucine, and isoleucine). The proteins were purified from the soluble fraction by affinity chromatography using Ni-Sepharose beads. The eluate was further purified using HiLoad 16/60 Superdex 200 (GE Healthcare). All purified proteins were concentrated and stored in 20 mM Tris–HCl buffer (pH 8.0) containing 200 mM NaCl.

### Size-exclusion chromatography

The molecular mass and oligomeric state of relevant proteins were characterized on an AKTA FPLC (GE Healthcare) using a Superdex™ 200 Increase column 10/300 GL (Cytiva, Lot 10323365). The column was equilibrated with 20 mM Tris–HCl, pH 8.0, and 200 mM NaCl and run at 0.7 ml/min at 16°C. A calibration curve for molecular size estimation was generated by individually loading thyroglobulin, gamma globulin, ovalbumin, myoglobin, and vitamin B12 on this analytical column and eluting under the same conditions.

### Crystallization and data collection

Initial crystallization trials were carried out with Crystal Screen, Crystal Screen 2, and PEG/Ion Screen reagent kits (Hampton Research) at 287 K by using the hanging-drop vapor-diffusion method. Each drop, consisting of 1 μl protein solution (10–30 mg/ml) and an equal volume of reservoir solution, was equilibrated against 200 μl reservoir solution. Further crystal optimization experiments were performed by systematic variation of the precipitant concentration and protein concentration and by testing the effects of additives. The best apo-SidN crystals were produced by mixing 1 μl of protein solution and an equal volume of reservoir solution containing 0.1 M sodium acetate (pH 4.6) and 2.4 M sodium chloride and incubating at 14°C. The best SeMet-SidN crystals were produced following the same protocol as that for native SidN except that the reservoir solution contained 0.1 M sodium acetate (pH 4.6) and 2.2 M sodium chloride. The crystals grew to their full dimensions in the final conditions after two weeks. The crystals were immersed briefly in a cryoprotectant solution consisting of 85% (*v*/*v*) reservoir solution and 15% (*v*/*v*) glycerol before flash-freezing with liquid nitrogen. Diffraction data were collected at beamline BL17U1 of Shanghai Synchrotron Radiation Facility (SSRF) and processed with the HKL2000 program suite (Otwinowski and Minor, 1997) or iMOSFLM (Battye et al., 2011) from the CCP4 program package.

### Structure determination

The initial phase was calculated using AutoSol, and an initial model was built using AutoBuild from PHENIX (Liebschner et al., 2019). We were able to trace most of the residues into an electron density map. Using the native data set and the initial model as a search coordinate, the structure of native SidN was determined by molecular replacement with the Phaser program (McCoy et al., 2007). The model was completed by iterative manual building in Coot (Emsley et al., 2010) and refined with REFMAC (Murshudov et al., 2011) and PHENIX (Liebschner et al., 2019). The final refined model contains one SidN molecule in the asymmetric unit and was refined to an *R* factor (*R*_free_) of 18.4% (23.2%). The structure contains all the residues except the recombinant 6-His tag and two C-terminal residues. The quality of the final model was evaluated using MolProbity (Chen et al., 2010). The statistics of data collection and refinement are summarized in Supplementary Table S1. The coordinates and structure factors have been deposited in the Protein Data Bank under the accession code 7YJI.

The DALI was used for structure similarity search (Holm and Rosenstrom, 2010). Amino acid sequences were aligned by Multalin (Corpet, 1988), and the structure-based sequence alignment was performed using ESPript (Robert and Gouet, 2014). All other structural figures were prepared with PyMOL(v0.99) (DeLano, 2002).

### Cell culture and plasmid construction

HEK293T, HeLa, A549, PC9, NIH3T3, and RAW 264.7 cells were cultured in Dulbecco's modified minimal Eagle's medium (VivaCell, C3113-0500) supplemented with 10% (*v*/*v*) fetal bovine serum (VivaCell, 04‐001-1A), 50 U/ml penicillin, and 50 mg/ml streptomycin (VivaCell, 03‐031-5B). All cells were grown at 37°C in a 5% CO_2_ humidified incubator and checked for potential mycoplasma contamination by the universal mycoplasma detection kit (Beyotime Biotechnology, C0297S).

For ectopic expression of proteins in mammalian cells, full-length SidN and mutants were cloned into pcDNA3.1 with an N-terminal EGFP tag, and other genes were inserted into pcDNA3.1 with an N-terminal Flag or HA tag. For the cytotoxicity test, genes were inserted into pCMV-Tet3G with an N-terminal EGFP tag. Plasmids were constructed by the homologous recombination method according to the instruction for the ClonExpress Ultra One Step Cloning Kit (Vazyme, C115-01). All plasmids and primers are listed in Supplementary Tables S2 and S3. Details about bacteria and cell lines are provided in Supplementary Table  S4.

### Transfection and co-IP

Plasmids were transfected into cells (80% confluence) using Lipo8000 (Beyotime, C0533) according to the manufacturer's protocol. After 24 h, cells were lysed in cell lysis buffer (Beyotime, P0013) at 4°C for 30 min. The supernatant of the lysate was collected after centrifugation at 4°C and 2500 rpm for 10 min and mixed with Protein G Agarose beads (Beyotime, P2053) that were pre-incubated with anti-GFP or anti-HA antibodies at 4°C for 4 h. After incubation overnight at 4°C, the beads were washed with pre-cold cell lysis buffer five times. Samples were resolved by sodium dodecyl sulfate–polyacrylamide gel electrophoresis (SDS–PAGE) followed by immunoblotting analysis with specific antibodies. The details of the antibodies are listed in Supplementary Table S4.

### Western blotting

Cells were lysed in radioimmunoprecipitation assay buffer (Beyotime, P0013B) at 4°C for 30 min and centrifuged at 12000 rpm. The soluble fraction was collected for SDS–PAGE, followed by immunoblotting analysis with antibodies against GFP, HA, β-actin, His, GAPDH, β-Tubulin, Lamin-B2, and Importin-13.

### Immunofluorescence

Cells were transfected with the indicated plasmids. After 24 h, cells were washed with phosphate-buffered saline (PBS), fixed in 4% paraformaldehyde at room temperature for 30 min, and then permeabilized with PBS–Triton X-100 (0.5%, *v/v*) for 10 min after three washes with PBS. Next, cells were incubated with antibodies (1:200/5% bovine serum albumin, antibody information is shown in Supplementary Table S4) at 4°C overnight. Subsequently, cells were incubated with appropriate fluorescence-labeled secondary antibodies at room temperature for 1 h. Finally, cells were stained with Hoechst 33342 (1:1000, Beyotime, C1025) and washed three times with PBS, and 20 μl fluorescence decay-resistant sealing tablets were added. Hoechst and immunol signals were analyzed with a fluorescence microscope (Leica SP8). Images were processed using ImageJ software.

### MTT assay

The cytotoxicity of SidN in HEK293T cells was measured by MTT assay. Briefly, cells were cultured in 6-well plates (80% confluence) and transfected with full-length SidN or mutants using Lipo8000. After 4 h, cells were digested by trypsin and seeded into 96-well plates at a density of 5 × 10^3^ cells/well. Twelve hours later, cells were induced by 100 ng/ml doxycycline for 6, 12, and 24 h. Next, 10 μl MTT solution (5 mg/ml) was added to each well and incubated for 4 h. Finally, the medium was removed, and 150 μl DMSO was used to dissolve the formed formazan in cells. After shaking at 60 rpm for 10 min at room temperature, the absorbance optical density values at the wavelength of 490 nm (OD490 nm) were determined by a multimode reader (SpectraMaxP1, Molecular Devices). The relative proliferation ratio was calculated as: OD490 nm of samples/OD490 nm of the EGFP group (without doxycycline induction) at 0 h.

### LC–MS/MS analysis

LC–MS/MS analysis was conducted at the Core Facility Center for Life Science, University of Science and Technology of China (Anhui, China). EGFP-tagged SidN protein was expressed in HEK293T cells, followed by IP with Protein G Agarose beads incubated with anti-GFP antibody, and the samples were separated by SDS–PAGE. After Coomassie brilliant blue staining, samples were excised and subjected to in-gel digestion with trypsin. Peptides were analyzed with LC–MS/MS with an EASY-nLC 1000 system (Thermo Fisher Scientific) and a Q Exactive mass spectrometer (Thermo Fisher Scientific). Proteome Discoverer 1.7 (Thermo Fisher Scientific) was used for protein identification.

### Surface sensing of translation assay

The level of protein translation in cells was detected by the surface sensing of translation assay (Schmidt et al., 2009). Briefly, the recombinant plasmids were transiently transfected into HEK293T cells using Lipo8000 (Beyotime, C0533) according to the manufacturer's protocol. After 24 h, puromycin (10 μg/ml) was added to the cell dishes, and after 10 min, the cells were washed three times in PBS. After cell lysis, proteins were separated by SDS–PAGE, and puromycin incorporation was detected by immunoblotting with a mouse monoclonal anti-puromycin antibody (Supplementary Table S4).

### Flow cytometric analysis of cell death

Cell death detection was performed according to the manufacturer's protocols for the Apoptosis and Necrosis Assay Kit (Beyotime, C1056) and the Annexin V–mCherry Apoptosis Detection Kit (Beyotime, C1069S). HEK293T cells were cultured in 6-well plates and transfected with the indicated plasmids. After 24 h, the cells were harvested by centrifugation at 1000× *g* for 5 min at 4°C. For the apoptosis and necrosis/pyroptosis assay, the cells were resuspended in 200 μl of 1× binding buffer containing 5 μl Hoechst and 5 μl PI and incubated for 20 min at room temperature. For Annexin V–mCherry apoptosis detection, 1 × 10^5^ cells were gently resuspended in 195 μl Annexin V–mCherry binding buffer and 5 μl Annexin V–mCherry and incubated at room temperature in the dark for 20 min. Finally, the cells were examined by flow cytometry (Gallios, Beckman) and analyzed by Kaluza analysis software.

### Knockdown of Importin-13

To inhibit Importin-13 expression, siRNAs targeting Importin-13 were synthesized by General Biol. The siImportin-13 sequences are 5′-UUCUUAUUCUCAAUGUUGGGA-3′ and 5′-CCAACAUUGAGAAUAAGAACC-3′. The negative control siRNA sequences are 5′-GCACUCGUCAACAUGAUUATT-3′ and 5′-UAAUCAUGUUGACGAGUGCTT-3′. HEK293T cells were cultured in 6-well plates and co-transfected with EGFP or EGFP-SidN plasmids and siRNAs (100 nM/ml) using Lipo8000 (Beyotime, C0533). After 24 h, proteins were resolved by SDS–PAGE and monitored by immunoblotting assays.

### Subcellular fractionation

The cytoplasmic and nuclear fractions were isolated according to the manufacturer's protocol for the Nuclear and Cytoplasmic Protein Extraction Kit (Beyotime, P0028).

### Statistics of abnormal nuclear structure

Nuclear structure was examined by confocal fluorescence microscopy. HEK293T cells were cultured in confocal dishes and transfected with pcDNA3.1-EGFP and pcDNA3.1-EGFP-SidN plasmids. After 24 h, cells were fixed and nuclei were stained with Hoechst. Then, cells were observed and photographed under a microscope. The ratio of abnormal nuclear structure in successfully transfected cells was calculated.

### Statistical analysis

Data are shown as mean ± standard deviation (SD) from three independent experiments and were evaluated using the GraphPad Prism Version 6.01 software. The significance of differences between groups was determined by Student's independent sample *t*-test. *P* < 0.05 was considered statistically significant.

## Supplementary Material

mjad036_Supplemental_Files

## Notes

**Author contributions:** J.G., X.Q., and H.G. conceived the study and designed the experiments. J.G., W.X., F.T., and X.C. performed experiments. J.G., X.Q., and H.G. analyzed the data. M.X., Q.Z., X.Y., N.Z., and J.M. provided expertise and methods. X.C., X.Q., and H.G. were responsible for funding acquisition. Q.Y. and X.Q. participated in manuscript writing. J.G. and H.G. wrote the paper with inputs from all authors.

## References

[bib1] Ahn J. , Jo I., Kang S.M. et al. (2019). Structural basis for lamin assembly at the molecular level. Nat. Commun. 10, 3757. 31434876 10.1038/s41467-019-11684-x PMC6704074

[bib2] Ashkenazy H. , Abadi S., Martz E. et al. (2016). ConSurf 2016: an improved methodology to estimate and visualize evolutionary conservation in macromolecules. Nucleic Acids Res. 44, W344–W350. 27166375 10.1093/nar/gkw408 PMC4987940

[bib3] Banga S. , Gao P., Shen X. et al. (2007). Legionella pneumophila inhibits macrophage apoptosis by targeting pro-death members of the Bcl2 protein family. Proc. Natl Acad. Sci. USA 104, 5121–5126. 17360363 10.1073/pnas.0611030104 PMC1829273

[bib4] Battye T.G. , Kontogiannis L., Johnson O. et al. (2011). iMOSFLM: a new graphical interface for diffraction-image processing with MOSFLM. Acta Crystallogr. D Biol. Crystallogr. 67, 271–281. 21460445 10.1107/S0907444910048675 PMC3069742

[bib5] Bond C.S. (2003). TopDraw: a sketchpad for protein structure topology cartoons. Bioinformatics 19, 311–312. 12538265 10.1093/bioinformatics/19.2.311

[bib6] Broz P. , Ruby T., Belhocine K. et al. (2012). Caspase-11 increases susceptibility to Salmonella infection in the absence of caspase-1. Nature 490, 288–291. 22895188 10.1038/nature11419 PMC3470772

[bib7] Bruggemann H. , Hagman A., Jules M. et al. (2006). Virulence strategies for infecting phagocytes deduced from the in vivo transcriptional program of Legionella pneumophila. Cell. Microbiol. 8, 1228–1240. 16882028 10.1111/j.1462-5822.2006.00703.x

[bib8] Chen V.B. , Arendall W.B., III, Headd J.J. et al. (2010). MolProbity: all-atom structure validation for macromolecular crystallography. Acta Crystallogr. D Biol. Crystallogr. 66, 12–21. 20057044 10.1107/S0907444909042073 PMC2803126

[bib9] Corpet F. (1988). Multiple sequence alignment with hierarchical clustering. Nucleic Acids Res. 16, 10881–10890. 2849754 10.1093/nar/16.22.10881 PMC338945

[bib10] Dechat T. , Gesson K., Foisner R. (2010). Lamina-independent lamins in the nuclear interior serve important functions. Cold Spring Harb. Symp. Quant. Biol. 75, 533–543. 21209392 10.1101/sqb.2010.75.018

[bib11] Dechat T. , Pfleghaar K., Sengupta K. et al. (2008). Nuclear lamins: major factors in the structural organization and function of the nucleus and chromatin. Genes Dev. 22, 832–853. 18381888 10.1101/gad.1652708 PMC2732390

[bib12] DeLano W.L. (2002). The PyMOL Molecular Graphics System. Palo Alto, CA: DeLano Scientific.

[bib13] Emsley P. , Lohkamp B., Scott W.G. et al. (2010). Features and development of Coot. Acta Crystallogr. D Biol. Crystallogr. 66, 486–501. 20383002 10.1107/S0907444910007493 PMC2852313

[bib14] Gajewska K.A. , Lescesen H., Ramialison M. et al. (2021). Nuclear transporter Importin-13 plays a key role in the oxidative stress transcriptional response. Nat. Commun. 12, 5904. 34625540 10.1038/s41467-021-26125-x PMC8501021

[bib15] Ge J. , Gong Y.N., Xu Y. et al. (2012). Preventing bacterial DNA release and absent in melanoma 2 inflammasome activation by a Legionella effector functioning in membrane trafficking. Proc. Natl Acad. Sci. USA 109, 6193–6198. 22474394 10.1073/pnas.1117490109 PMC3341053

[bib16] Holm L. , Rosenstrom P. (2010). Dali server: conservation mapping in 3D. Nucleic Acids Res. 38, W545–W549. 20457744 10.1093/nar/gkq366 PMC2896194

[bib17] Kapinos L.E. , Schumacher J., Mucke N. et al. (2010). Characterization of the head-to-tail overlap complexes formed by human lamin A, B1 and B2 ‘half-minilamin’ dimers. J. Mol. Biol. 396, 719–731. 20004208 10.1016/j.jmb.2009.12.001

[bib18] Krissinel E. , Henrick K. (2007). Inference of macromolecular assemblies from crystalline state. J. Mol.Biol. 372, 774–797. 17681537 10.1016/j.jmb.2007.05.022

[bib19] Laguna R.K. , Creasey E.A., Li Z. et al. (2006). A Legionella pneumophila-translocated substrate that is required for growth within macrophages and protection from host cell death. Proc. Natl Acad. Sci. USA 103, 18745–18750. 17124169 10.1073/pnas.0609012103 PMC1656969

[bib20] Liebschner D. , Afonine P.V., Baker M.L. et al. (2019). Macromolecular structure determination using X-rays, neutrons and electrons: recent developments in Phenix. Acta Crystallogr. D Struct. Biol. 75, 861–877. 31588918 10.1107/S2059798319011471 PMC6778852

[bib21] Lindenboim L. , Zohar H., Worman H.J. et al. (2020). The nuclear envelope: target and mediator of the apoptotic process. Cell Death Discov. 6, 29. 32351716 10.1038/s41420-020-0256-5 PMC7184752

[bib22] McCoy A.J. , Grosse-Kunstleve R.W., Adams P.D. et al. (2007). Phaser crystallographic software. J. Appl. Crystallogr. 40, 658–674. 19461840 10.1107/S0021889807021206 PMC2483472

[bib23] Mingot J.M. , Kostka S., Kraft R. et al. (2001). Importin 13: a novel mediator of nuclear import and export. EMBO J. 20, 3685–3694. 11447110 10.1093/emboj/20.14.3685 PMC125545

[bib24] Mondino S. , Schmidt S., Rolando M. et al. (2020). Legionnaires' disease: state of the art knowledge of pathogenesis mechanisms of Legionella. Annu. Rev. Pathol. Mech. Dis. 15, 439–466. 10.1146/annurev-pathmechdis-012419-032742 31657966

[bib25] Murshudov G.N. , Skubak P., Lebedev A.A. et al. (2011). REFMAC5 for the refinement of macromolecular crystal structures. Acta Crystallogr. D Biol. Crystallogr. 67, 355–367. 21460454 10.1107/S0907444911001314 PMC3069751

[bib26] Nogueira C.V. , Lindsten T., Jamieson A.M. et al. (2009). Rapid pathogen-induced apoptosis: a mechanism used by dendritic cells to limit intracellular replication of Legionella pneumophila. PLoS Pathog. 5, e1000478. 19521510 10.1371/journal.ppat.1000478 PMC2689937

[bib27] Otwinowski Z. , Minor W. (1997). Processing of X-ray diffraction data collected in oscillation mode. Meth. Enzymol. 276, 307–326. 10.1016/S0076-6879(97)76066-X 27754618

[bib28] Qiu J. , Luo Z.Q. (2017). Legionella and Coxiella effectors: strength in diversity and activity. Nat. Rev. Microbiol. 15, 591–605. 28713154 10.1038/nrmicro.2017.67

[bib29] Robert X. , Gouet P. (2014). Deciphering key features in protein structures with the new ENDscript server. Nucleic Acids Res. 42, W320–W324. 24753421 10.1093/nar/gku316 PMC4086106

[bib30] Schmidt E.K. , Clavarino G., Ceppi M. et al. (2009). SUnSET, a nonradioactive method to monitor protein synthesis. Nat. Methods 6, 275–277. 19305406 10.1038/nmeth.1314

[bib31] Schroeder G.N. (2017). The toolbox for uncovering the functions of Legionella Dot/Icm type IVb secretion system effectors: current state and future directions. Front. Cell. Infect. Microbiol. 7, 528. 29354599 10.3389/fcimb.2017.00528 PMC5760550

[bib32] Sen Gupta A. , Sengupta K. (2017). Lamin B2 modulates nucleolar morphology, dynamics, and function. Mol. Cell. Biol. 37, e00274–e00217. 10.1128/MCB.00274-17 PMC5705821 28993479

[bib33] Shimi T. , Pfleghaar K., Kojima S. et al. (2008). The A- and B-type nuclear lamin networks: microdomains involved in chromatin organization and transcription. Genes Dev. 22, 3409–3421. 19141474 10.1101/gad.1735208 PMC2607069

[bib34] Speir M. , Vogrin A., Seidi A. et al. (2017). Legionella pneumophila strain 130b evades macrophage cell death independent of theeffector SidF in the absence of flagellin. Front. Cell. Infect. Microbiol. 7, 35. 28261564 10.3389/fcimb.2017.00035 PMC5311068

[bib35] Vogel J.P. , Andrews H.L., Wong S.K. et al. (1998). Conjugative transfer by the virulence system of Legionella pneumophila. Science 279, 873–876. 9452389 10.1126/science.279.5352.873

[bib36] Zhu W. , Hammad L.A., Hsu F. et al. (2013). Induction of caspase 3 activation by multiple Legionella pneumophila Dot/Icm substrates. Cell. Microbiol. 15, 1783–1795. 23773455 10.1111/cmi.12157 PMC3797225

