# Supplementary material for: The bacterial effector SidN/Lpg1083 promotes cell death by targeting Lamin-B2
Source: J Mol Cell Biol. 2023 May 30;15(5):mjad036. doi: 10.1093/jmcb/mjad036 (PMC10729856; doi:10.1093/jmcb/mjad036)
Supplement: mjad036_Supplemental_Files [file mjad036_supplemental_files.zip › Supplemental_Materials-final-v2.pdf]

Supplementary materials

Supplemental Figures

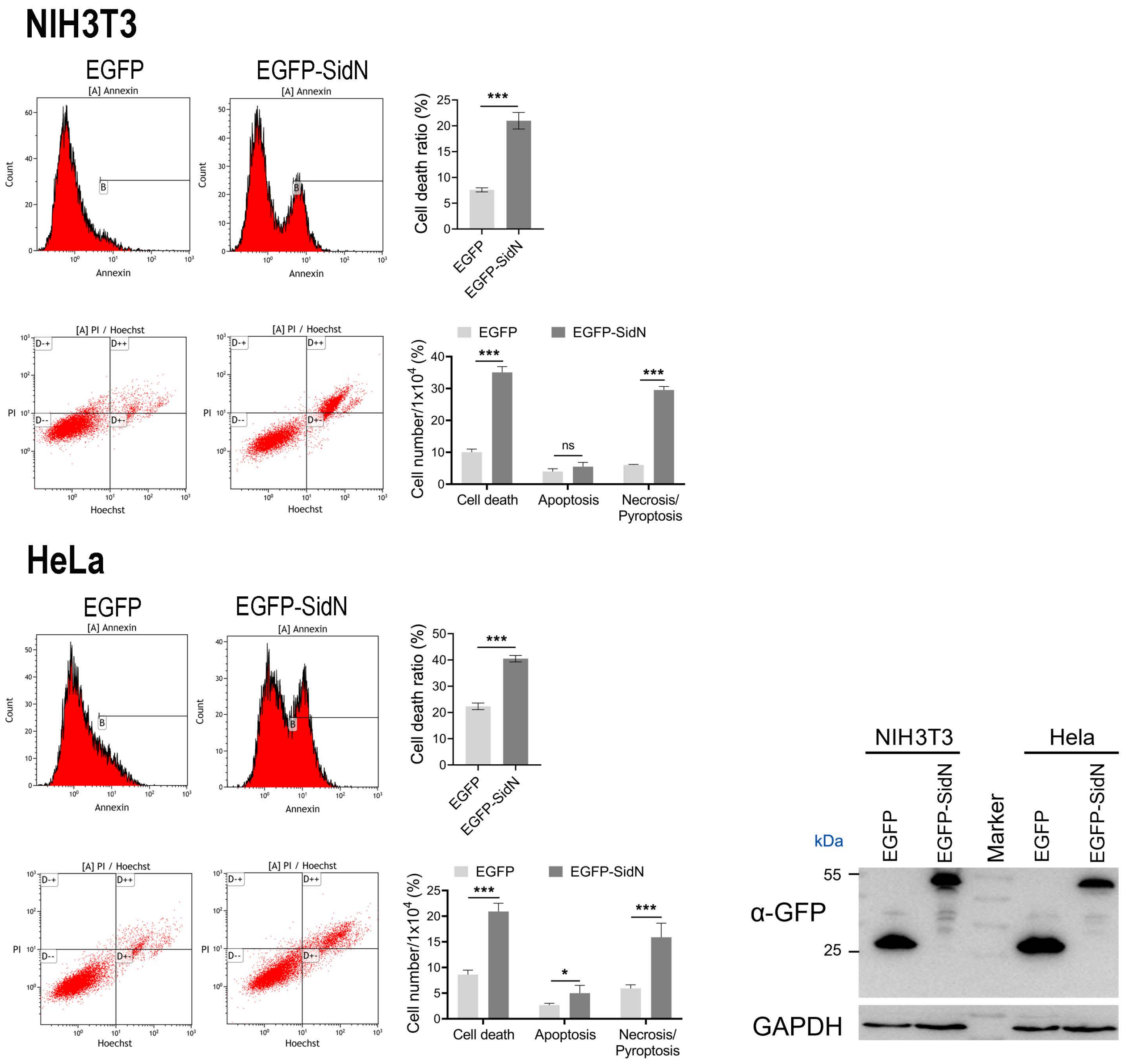

Supplementary Figure S1

SidN exhibits toxicity to NIH3T3 and Hela cells.

Representative flow cytometry results from Annexin V-mCherry Apoptosis Detection and Apoptosis and Necrosis/pyroptosis assay. The ratio of cell death was calculated by flow cytometry from three independent experiments. The ratio of apoptosis, necrosis/pyroptosis and total cell death were calculated by flow-cytometric from three independent experiments. Data shown as the mean ± SD, ***$P < 0.001$ in *t-test*. The supernatants of NIH3T3 and Hela cells lysates were subjected to detect EGFP, EGFP-SidN and GAPDH expression by western blot assay.

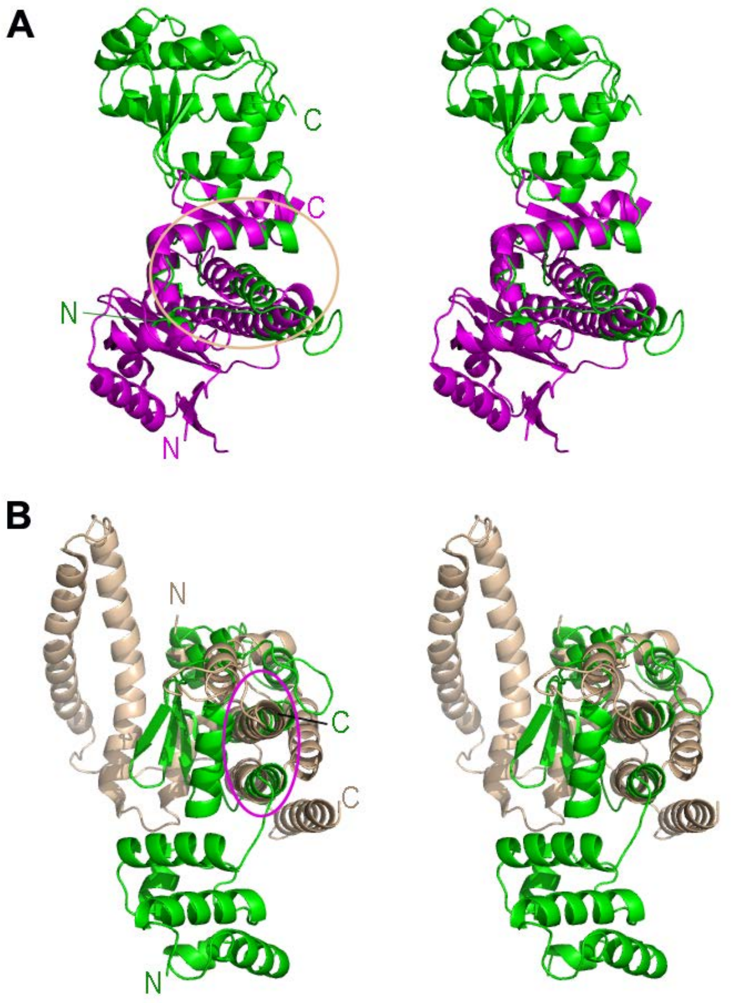

Supplementary Figure S2

Superposition of SidN and the best match structures (Stereo view).

(**A**) Superposition of SidN (green) and 2QLZ (magenta).

(**B**) Superposition of SidN (green) and 5X41 (wheat).

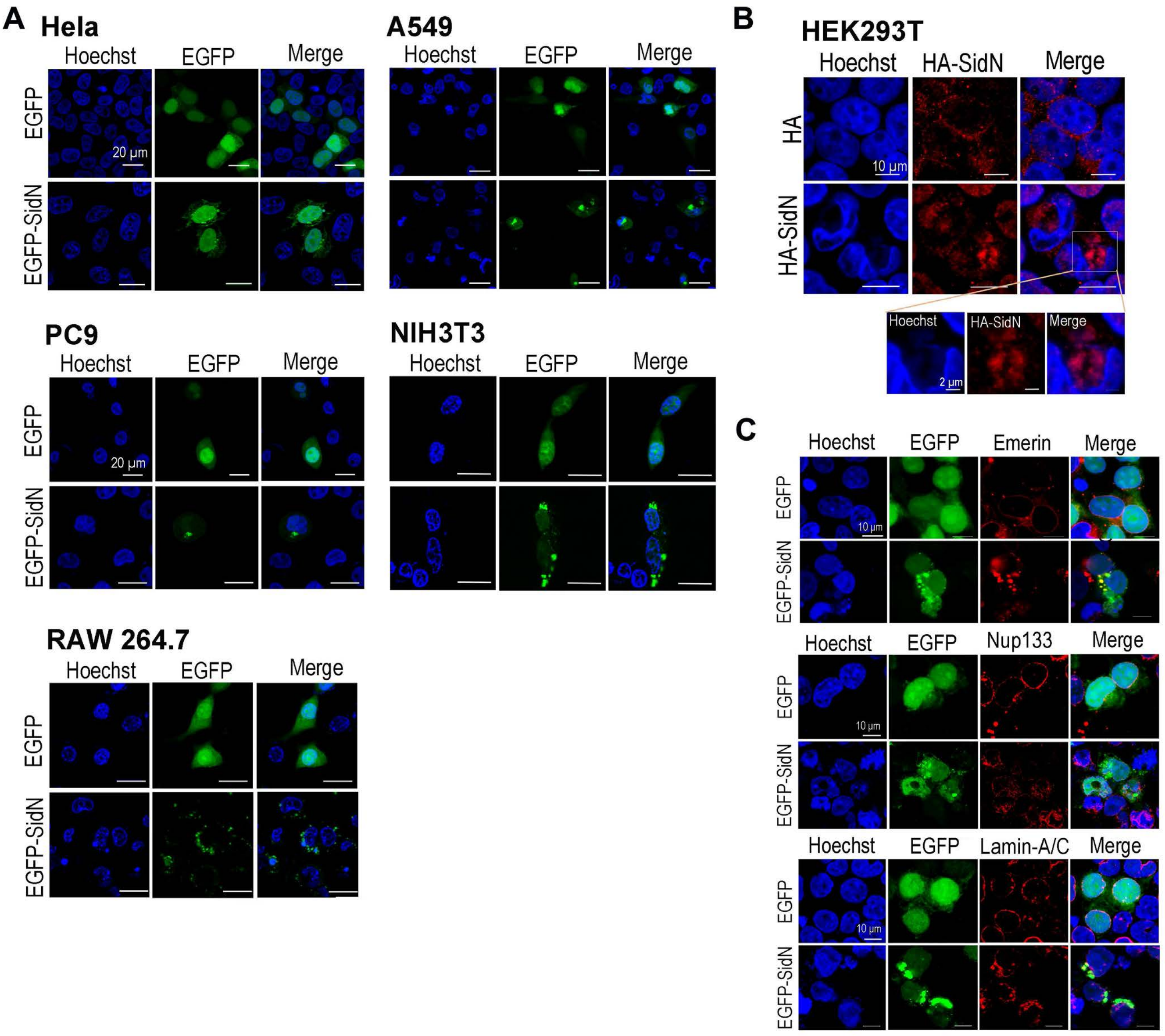

Supplementary Figure S3

SidN co-localizes with the morphologically altered nuclear envelope.

(**A**) Confocal microscopy images of the intracellular localization after transfection for 24 h with EGFP or EGFP-SidN in HeLa, A549, RAW 264.7, PC9 and NIH3T3 cells. Green fluorescence indicates the position of EGFP or EGFP-SidN, and cell nuclei were visualized with Hoechst stain (blue).

(**B**) Confocal microscopy images of the intracellular localization after transfection for 24 h with HA or HA-SidN in HEK293T cells. Cell nuclei were visualized with Hoechst stain (blue) and Cy3 conjugated IgG anti-rabbit antibodies and HA rabbit antibodies were used to display HA-Importin-13 (red).

(**C** Localization of SidN and in-situ Emerin, Nup133 and Lamin-A/C. Green fluorescence indicates the position of EGFP or EGFP-SidN, cell nuclei were visualized with Hoechst stain (blue). Cy3 conjugated IgG anti-rabbit antibodies and Emerin, Nup133 or Lamin-A/C rabbit special antibodies were used to display the in-situ Emerin, Nup133 and Lamin-A/C (red).

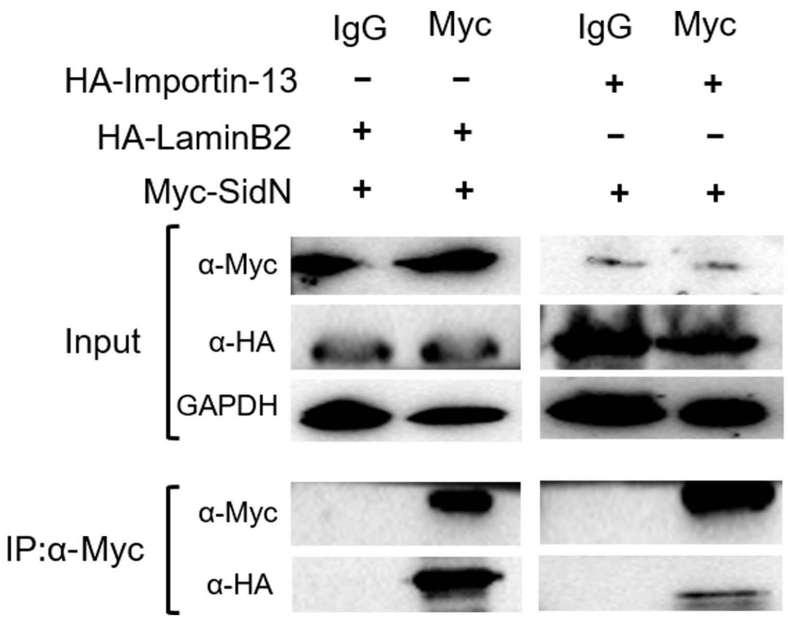

Supplementary Figure S4

Myc-SidN interacts with Lamin-B2 and Importin-13.

Co-immunoprecipitation of Myc-SidN and HA-Lamin B2 or HA-Importin-13. The lysates of HEK293T cells transfected with the indicated plasmid combinations were immunoprecipitated with a Myc-specific antibody, and the remaining part were probed with both Myc and HA antibodies.

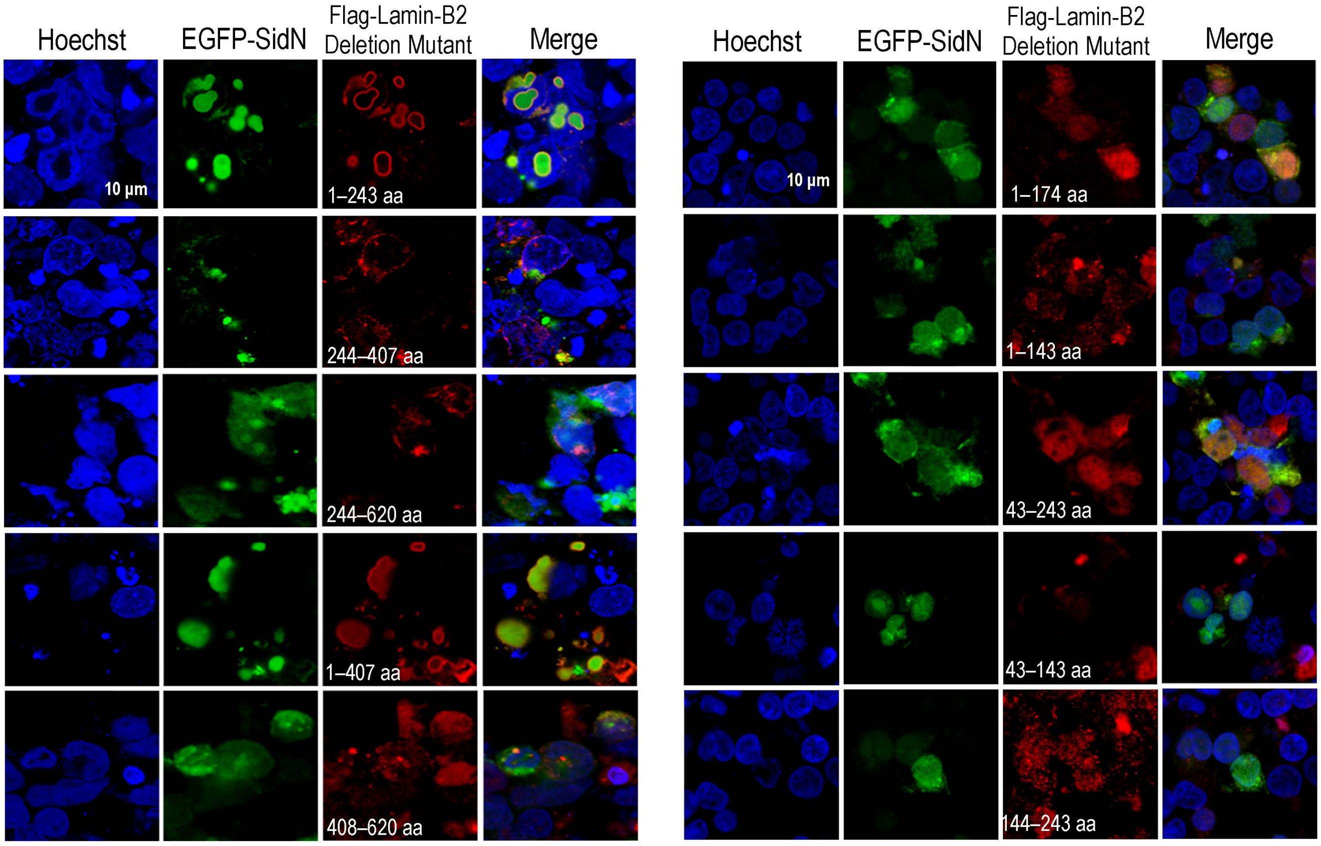

Supplementary Figure S5

Co-localization of SidN and Lamin-B2 deletion mutants.

Green fluorescence indicates the position of EGFP-SidN, cell nuclei were visualized with Hoechst stain (blue), and Cy3 conjugated IgG anti-rabbit antibodies and HA rabbit antibodies were used to show the position of HA-Lamin-B2 mutants.

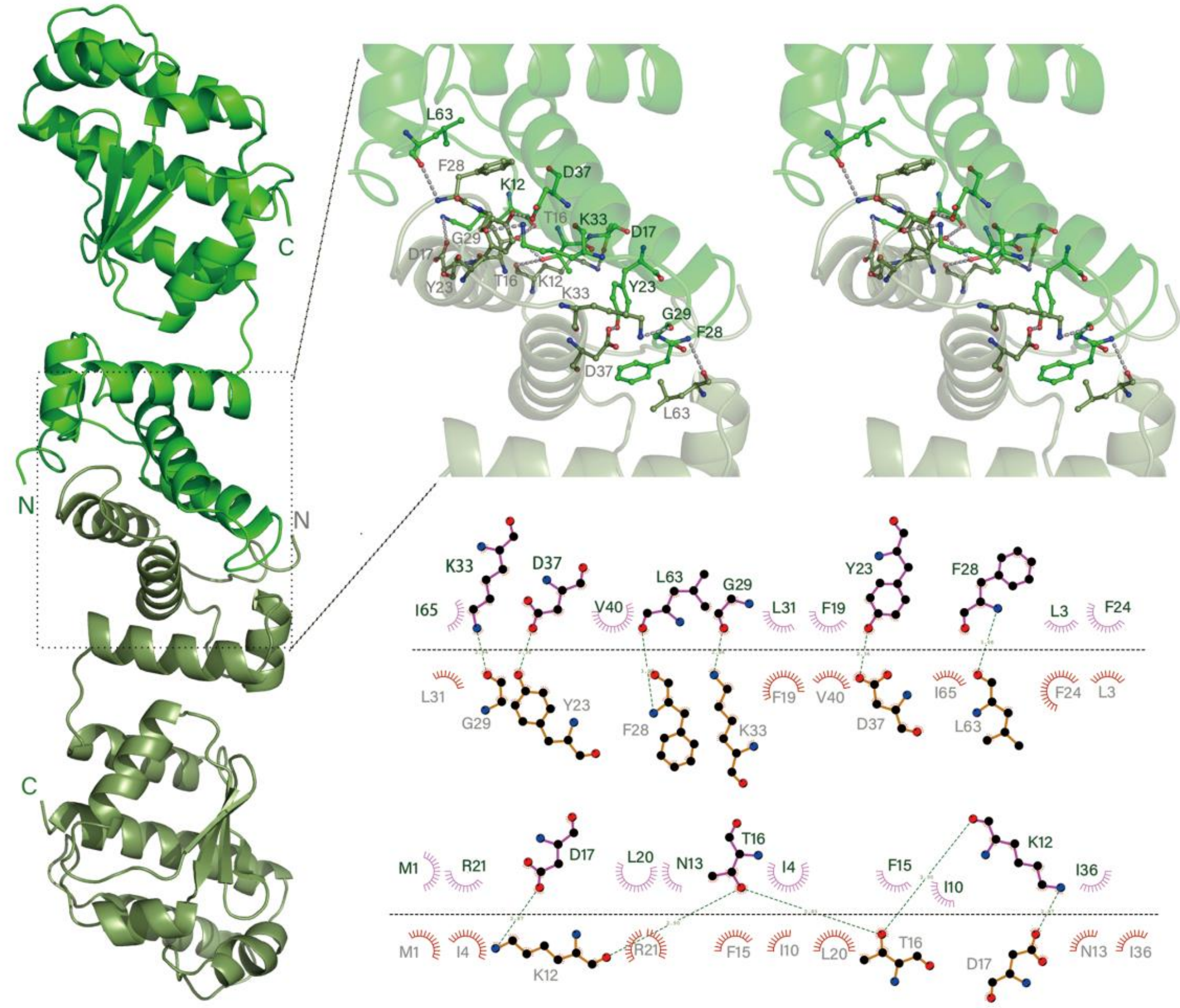

Supplementary Figure S6

SidN forms a dimer with its crystallographic symmetry mate.

In (left panel), ribbon representation of the dimer organization of SidN. In (top right panel), closed-up view of the interactions of SidN and its neighboring monomer. The relevant residues are labeled and shown in stick form. Dotted lines, hydrogen bonds. In (bottom right panel), Ligplot diagram illustrating the molecular interactions in a SidN dimer. Hydrogen bonding and hydrophobic interactions were illustrated using Ligplot (Laskowski and Swindells, 2011).

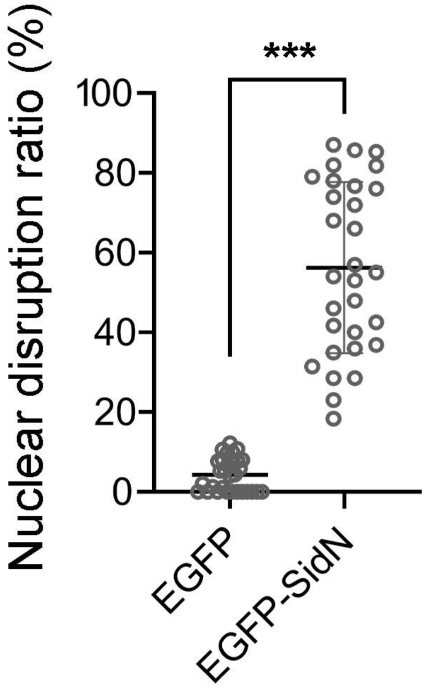

Supplementary Figure S7

The statistics of the abnormal nuclear structure induced by SidN.

The ratio was calculated by thirty confocal images of different horizons and three independent experiments. Data are shown as the mean ± SD, ****P* < 0.001 in *t-test*.

Supplemental Video

Supplementary Video S1
Time-lapse images of EGFP-SidN localization.

Live-cell fluorescence microscopy of HEK293T cells expressing EGFP-SidN (green). Cell nuclei were visualized with Hoechst stain (blue). Images were taken every 20 min. Scale bar, 20 μm.

Supplemental Tables

Supplementary Table S1. Data collection and refinement statistics

	SeMet-SidN	Native SidN
**Data collection**		
SSRF beamline	BL17U	BL17U
Wavelength(Å)	0.97791	0.97931
Space group	I422	I422
Molecules/ASU	1	1
Cell parameters		
a/b/c (Å)	92.22/92.22/135.91	93.66/93.66/136.26
Resolution range (Å)	50-2.8(2.85-2.80)	77.18-2.10(2.21-2.10)
No. of unique reflections	7537(372)	17123(2590)
$R_{p.i.m.}$[a] (%)	1.9(8.8)	4.1(28.9)
Average I/σ(I)	40.2(6.5)	12.3(2.9)
$CC_{1/2}$	(0.98)	0.997(0.843)
Completeness (%)	100(100)	94.7(100)
Redundancy	22.4(23.1)	15.7(16.1)
**Refinement**		
PDB entry		7YJI
Resolution limits (Å)		19.3-2.1
No. of reflections		17077(1762)
R factor [b] (%)		18.2(23.5)
Free R factor [c] (%)		23.3(28.9)
No. of protein atoms		1866
No. of solvent molecules		104
rmsd [d] in bond lengths (Å)		0.008
rmsd in bond angles (°)		0.83
B-factor (Å^2)		
macromolecules		44.53
Waters		44.29
Ramachandran plot [e] (%)		
favored/disallowed		96.41/0
MolProbity score / Clash score		1.40/3.72

Values in parentheses refer to the highest resolution shell.

[a] $R_{p.i.m} = \sum_{hkl} [1/(n_{hkl} - 1)]^{1/2} \sum_i |I_i(hkl) - \langle I(hkl)\rangle| / \sum_{hkl} \sum_i I_i(hkl)$, where n_{hkl} is the number of observations of reflection *hkl.*

[b] R-factor $=\sum h \mid |F_{obs}| - |F_{calc}| \mid / \sum |F_{obs}|$, where $|F_{obs}|$ and $|F_{calc}|$ are the observed and calculated structure factor amplitudes, respectively. Summation includes all reflections used in the refinement.

[c] Free R factor $= \sum \mid |F_{obs}| - |F_{calc}| \mid / \sum |F_{obs}|$, evaluated for a randomly chosen subset of 5% of the diffraction data not included in the refinement.

[d] Root-mean square-deviation from ideal values.

[e] Categories were defined by Molprobity.

Supplementary Table S2. Plasmids used in this study.

Plasmids	Genotype	Purpose	Reference or source
pET-28a	Cloning vector, Kan ^{r}	Cloning	Novagen
pET-28a-His-SidN	Cloning vector encoding Lpg1083, Kan ^{r}	Protein expression & purification	This study
pET-28a-His-SidN$^{T55A/Y57A/R80A}$	Cloning vector encoding Lpg1083 catalytic T55A/Y57A/R80A mutant, Kan ^{r}	Protein expression & purification	This study
pCDNA3.1(+)	Cloning vector, Amp ^{r}	Cloning	Addgene
pCDNA3.1(+)-HA	Cloning vector, Amp ^{r}	Cloning	This study
pCDNA3.1(+)-EGFP-SidN	Cloning vector encoding EGFP-Lpg1083, Amp ^{r}	Transfection	This study
pCDNA3.1(+)-Myc-SidN	Cloning vector encoding Myc-Lpg1083, Amp ^{r}	Transfection	This study
pCDNA3.1(+)-EGFP-SidN$_{1-82}$	Encoding wild type EGFP-Lpg1083 from 1-82 aa, Amp ^{r}	Transfection	This study
pCDNA3.1(+)-EGFP-SidN$_{83-227}$	Encoding wild type EGFP-Lpg1083 from 83-227 aa, Amp ^{r}	Transfection	This study
pCDNA3.1(+)-EGFP-SidN^{F19D}	Encoding EGFP-Lpg1083 F19D mutant, Amp ^{r}	Transfection	This study
pCDNA3.1(+)-EGFP-SidN$^{F19D/V40D}$	Encoding EGFP-Lpg1083 F19D and V40D mutant, Amp ^{r}	Transfection	This study
pCDNA3.1(+)-EGFP-SidN$^{T55A/Y57A/R80A}$	Encoding EGFP-Lpg1083 T55A, Y57A and R80A mutant, Amp ^{r}	Transfection	This study
pCDNA3.1(+)-HA-Lamin-B2	Cloning vector encoding Flag-HA-Lamin-B2, Amp ^{r}	Transfection	This study
pCDNA3.1(+)-HA-Lamin-B2$_{1-243}$	Encoding wild type Flag-HA-Lamin-B2 from 1 to 243 aa, Amp ^{r}	Transfection	This study
pCDNA3.1(+)-HA-Lamin-B2$_{244-407}$	Encoding wild type Flag-HA-Lamin-B2 from 244 to 407 aa, Amp ^{r}	Transfection	This study
pCDNA3.1(+)-HA-Lamin-B2$_{244-620}$	Encoding wild type Flag-HA-Lamin-B2 from 244 to 620 aa, Amp ^{r}	Transfection	This study
pCDNA3.1(+)-HA-Lamin-B2$_{1-407}$	Encoding wild type Flag-HA-Lamin-B2 from 1 to 407 aa, Amp ^{r}	Transfection	This study
pCDNA3.1(+)-HA-Lamin-B2$_{408-620}$	Encoding wild type Flag-HA-Lamin-B2 from 480 to 620 aa, Amp ^{r}	Transfection	This study
pCDNA3.1(+)-HA-Lamin-B2 $_{1-174}$	Encoding wild type Flag-HA-Lamin-B2 from 1 to 174 aa, Amp ^{r}	Transfection	This study
pCDNA3.1(+)-HA-Lamin-B2$_{1-143}$	Encoding wild type Flag-HA-Lamin-B2 from 1 to 143aa, Amp ^{r}	Transfection	This study
pCDNA3.1(+)-HA-Lamin-B2$_{43-243}$	Encoding wild type Flag-HA-Lamin-B2 from 43 to 243 aa, Amp ^{r}	Transfection	This study

pCDNA3.1(+)-HA-Lamin-B2 $_{43\text{-}143}$	Encoding Flag-HA-Lamin-B2 from 43 to 143 aa, Amp ^{r}	Transfection	This study
pCDNA3.1(+)-Flag-HA-Lamin-B2 $_{144\text{-}243}$	Encoding Flag-HA-Lamin-B2 from 144 to 243 aa, Amp ^{r}	Transfection	This study
pCDNA3.1(+)-Flag-HA-Lamin-B2 $_{43\text{-}174}$	Encoding Flag-HA-Lamin-B2 from 43 to 174 aa, Amp ^{r}	Transfection	This study
pCDNA3.1(+)-Lamin-B2-6×His	Encoding Lamin-B2-6×His Amp ^{r}	Transfection	This study
pCDNA3.1(+)-HA-Importin 13	Encoding Flag-HA-Importin 13, Amp ^{r}	Transfection	This study

Supplementary Table S3. List of primers used in this study.

ID	Name	Sequence (5' to 3')
1	EGFP forw. (pCDNA3.1(+)-EGFP-SidN)	CTTGGTACCGAGCTCGGATCATGGTGAGCAAGGGCGAGGAGCTGTTCACC
2	EGFP rev. (pCDNA3.1(+)-EGFP-SidN)	ACCTCCGCTACCTGTATCAGCTCCCTTGTACAGCTCGTCCATGCCGAGAG
3	SidN forw. (pCDNA3.1(+)-EGFP-SidN)	GATACAGGTAGCGGAGGTATGGCGCTAATTGATCAAATTACTACGATTAATAAG
4	SidN rev. (pCDNA3.1(+)-EGFP-SidN)	AAACGGGCCCTCTAGACTCGATTATATCCCTATGTGTGATTTCACCATAG
5	SidN forw. (pET-28a-His-SidN)	GGAATTCCATATGATGGCGCTAATTGATCAAATTACTAC
6	SidN rev. (pET-28a-His-SidN)	CCGCTCGAGTTATATCCCTATGTGTGATTTCACCATAG
7	Lpg1083 F19D forw.	TTACCGACGATGACCTGCGTAAATATTTTGAACTAGGTTT
8	Lpg1083 F19D rev.	TTTACGCAGGTCATCGTCGGTAAATTCATTCTTATTAATC
9	Lpg1083 V40D forw.	CACGATATTGATTTACTCGATTATTATCTTG
10	Lpg1083 V40D rev.	ATCGAGTAAATCAATATCGTGTTTAGATAAAG
11	Lpg1083 R80A forw.	CTACCTGGCATATGAAAATAATTCCATATC
12	Lpg1083 R80A rev.	TTCATATGCCAGGTAGGACTCCATT
13	Lpg1083 T55A forw.	TAATGGAAAAGCAAATTATGAAATTTCTTC
14	Lpg1083 T55A rev.	CATAATTTGCTTTTCCATTAAATAAGTCTGA
15	Lpg1083 T57A forw.	ACAAATGCTGAAATTTCTTCTCTTCTGACGATAAC
16	Lpg1083 T57A rev.	GAAATTTCAGCATTTGTTTTTCCATTAAATAAGTCTG
17	Lamin-B2 forw.	ATGAGCCCGCCGAGCCCGGGCCGCC
18	Lamin-B2 rev.	TCACATCACGTAGCAGCCTCTTGAGG
19	Lamin-B2$_{1\text{-}243}$ forw.	ATGAGCCCGCCGAGCCCGGGCCGCC
20	Lamin-B2$_{1\text{-}243}$ rev.	TTACTCCACCAGGCGCCGCTCGTGCC
21	Lamin-B2$_{244\text{-}407}$forw.	ATGGTGGACAGCAGCCGGCAGCAG
22	Lamin-B2$_{244\text{-}407}$rev.	TTAGCTGGGGGACAGCTTCAGCCTCTC
23	Lamin-B2$_{244\text{-}620}$ forw.	ATGGTGGACAGCAGCCGGCAGCAG
24	Lamin-B2$_{244\text{-}620}$ rev.	TCACATCACGTAGCAGCCTCTTGAGG
25	Lamin-B2$_{1\text{-}407}$ forw.	ATGAGCCCGCCGAGCCCGGGCCGCC
26	Lamin-B2$_{1\text{-}407}$ rev.	TTAGCTGGGGGACAGCTTCAGCCTCTC
27	Lamin-B2$_{408\text{-}620}$ forw.	ATGCCATCCTCGCGCGTCACCGTCTC
28	Lamin-B2$_{408\text{-}620}$ rev.	CACATCACGTAGCAGCCTCTTGAGG
29	Lamin-B2$_{1\text{-}174}$ forw.	ATGAGCCCGCCGAGCCCGGGCCGCC
30	Lamin-B2$_{1\text{-}174}$ rev.	TTACTCCAGGCCGCGCTTGTCGCTGAG
31	Lamin-B2$_{1\text{-}143}$ forw.	ATGAGCCCGCCGAGCCCGGGCCGCC
32	Lamin-B2$_{1\text{-}143}$ rev.	TTACGTAAGCTCGCCCTCCCTCTT

33	Lamin-B2$_{43\text{-}243}$ forw.	ATGCGGCTGCAGGAGAAGGAGGAGC
34	Lamin-B2$_{43\text{-}243}$ rev.	TTACTCCACCAGGCGCCGCTCGTGCC
35	Lamin-B2$_{43\text{-}143}$ forw.	ATGCGGCTGCAGGAGAAGGAGGAGC
36	Lamin-B2$_{43\text{-}143}$ rev.	TTACGTAAGCTCGCCCTCCCTCTT
37	Lamin-B2$_{144\text{-}243}$ forw.	ATGGTGGCCCAGGGCCGTGTGAA
38	Lamin-B2$_{144\text{-}243}$ rev.	TTACTCCACCAGGCGCCGCTCGTGCC
39	Lamin-B2$_{43\text{-}174}$ forw.	ATGCGGCTGCAGGAGAAGGAG
40	Lamin-B2$_{43\text{-}174}$ rev.	TTACAGGCCGCGCTTGTCG
41	Importin-13 forw.	ATGGAGCGGCGGGAGGAGCAGCCGG
42	Importin-13 rev.	TCAGTAGTCAGCTGTGTAATCTGTGC

Supplementary Table S4. Reagent and resource used in this study.

REAGENT or RESOURCE	SOURCE	IDENTIFIER Cat：
**Antibodies**		
Mouse monoclonal anti-GFP	Proteintech	Cat: # 66002-1-Ig
Rabbit Polyclonal anti-GFP	Proteintech	Cat: # 50430-2-AP
Rabbit Polyclonal anti-HA	Proteintech	Cat: # 51064-2-AP
Mouse monoclonal anti-HA	Proteintech	Cat: # 66006-2-Ig
Mouse monoclonal anti-MYC	Proteintech	Cat: # 60003-2-Ig
Rabbit Polyclonal anti-Lamin B2	Proteintech	Cat: # 10895-1-AP
Rabbit Polyclonal anti- Importin 13	Proteintech	Cat: # 11696-2-AP
Rabbit Polyclonal anti-Emerin	Proteintech	Cat: # 10351-1-AP
Rabbit Polyclonal anti-NUP133	Proteintech	Cat: # 12405-1-AP
Rabbit Polyclonal anti-LaminA/C	Proteintech	Cat: # 10298-1-AP
Mouse monoclonal anti-Puromycin	MERCK	Cat: # ZMS1016
Cy3-labeled Goat Anti-Rabbit IgG	Beyotime	Cat: # A0516
Cy3-labeled Goat Anti- Mouse IgG	Beyotime	Cat: # A 0507
Mouse monoclonal anti-GAPDH	Proteintech	Cat: # 60004-1-Ig
Mouse monoclonal anti-β-Actin	Proteintech	Cat: # 66009-1-Ig
Rabbit Polyclonal anti-β-Tubulin	Proteintech	Cat: # 10094-1-AP
Rabbit Polyclonal anti-Histone-H3	Proteintech	Cat: # 17168-1-AP
HRP-conjugated Affinipure Goat Anti-Rabbit IgG(H+L)	Proteintech	Cat: # SA00001-2
HRP-conjugated Affinipure Goat Anti-Mouse IgG(H+L)	Proteintech	Cat: # SA00001-1
Caspase 3/p17/p19 Monoclonal antibody	Proteintech	Cat: # 66470-2-Ig
GSDMD Monoclonal antibody	Proteintech	Cat: #66387-1-Ig
**Bacterial and Cell Lines**		
E. coli DH5α	TSINGKE	Cat: # TSC-C14
E. coli BL21(DE3) Rosetta	Sigama	Cat: # 70954
HEK293T	ATCC	Cat: # BFN60810479
Hela	ATCC	Cat: # BFN60700111
A549	ATCC	Cat: # BFN608007142
PC9	ATCC	Cat: # BFN60800699
RAW 264.7	ATCC	Cat: # BFN60807564
**Chemicals, Peptides, and Recombinant Proteins**		
IPTG	Sigama	CAS:367-93-1
Selenomethionine (Se-Met)	Sigama	Cat: # 1611955
Nickel-NTA resin	Thermo Scientific™	Cat: # 88221
